# Structure and function of atypically coordinated enzymatic mononuclear non-heme-Fe(II) centers

**DOI:** 10.1016/j.ccr.2012.04.028

**Published:** 2013-01-15

**Authors:** Daniela Buongiorno, Grit D. Straganz

**Affiliations:** Institute of Biotechnology and Biochemical Engineering, Graz University of Technology, Petersgasse 12 A-8010 Graz, Austria

**Keywords:** 1,3-bis(2-pyridylimino)isoindoline, ind, 2OH-1,3-Ph_2_PD, 2-hydroxy-1,3-diphenylpropanedione, 6-Ph_2_TPA, N,N-bis[(6-phenyl-2-pyridyl)methyl]-N-[(2-pyridyl)-methyl]amine, α-KG, α-ketoglutarate, acac, acetylacetone (2,4-pentanedione), ADO, cysteamine dioxygenase, AO, apocarotenoid 15,15′-oxygenase, ARD, aci-reductone dioxygenase, BsQDO, quercetin 2,3-dioxygenase from *Bacillus subtilis*, CarOs, carotenoid oxygenases, CD, circular dichroism, CDO, cysteine dioxygenase, CGDO, 5-chloro-gentisate 1,2-dioxygenase, CS2, clavaminate synthase, DFT, density functional theory, Dke1, diketone dioxygenase, EPR, electron paramagnetic resonance, EXAFS, extended X-ray absorption fine structure spectroscopy, fla, flavonolate, GDO, gentisate 1,2-dioxygenase, HADO, 3-hydroxyanthranilate 3,4-dioxygenase, HGDO, homogentisate 1,2-dioxygenase, HNDO, hydroxy-2-naphthoate dioxygenase, MCD, magnetic circular dichroism, MNHEs, mononuclear non-heme-Fe(II) dependent enzymes, NRP, nonribosomal peptide, OTf-, trifluormethanesulfonate, PDB, protein data bank, QDO, quercetin 2,3-dioxygenase, SDO, salicylate 1,2-dioxygenase, TauD, taurine hydroxylase, XAS, X-ray absorption spectroscopy, Enzyme catalysis, Dioxygen activation, Dioxygenase, Facial triad, Metal binding motif, Structure–function relationships

## Abstract

Mononuclear, non-heme-Fe(II) centers are key structures in O_2_ metabolism and catalyze an impressive variety of enzymatic reactions. While most are bound via two histidines and a carboxylate, some show a different organization. A short overview of atypically coordinated O_2_ dependent mononuclear-non-heme-Fe(II) centers is presented here Enzymes with 2-His, 3-His, 3-His-carboxylate and 4-His bound Fe(II) centers are discussed with a focus on their reactivity, metal ion promiscuity and recent progress in the elucidation of their enzymatic mechanisms. Observations concerning these and classically coordinated Fe(II) centers are used to understand the impact of the metal binding motif on catalysis.

## Graphical abstract

**Graphical abstract:**

**Figure:**
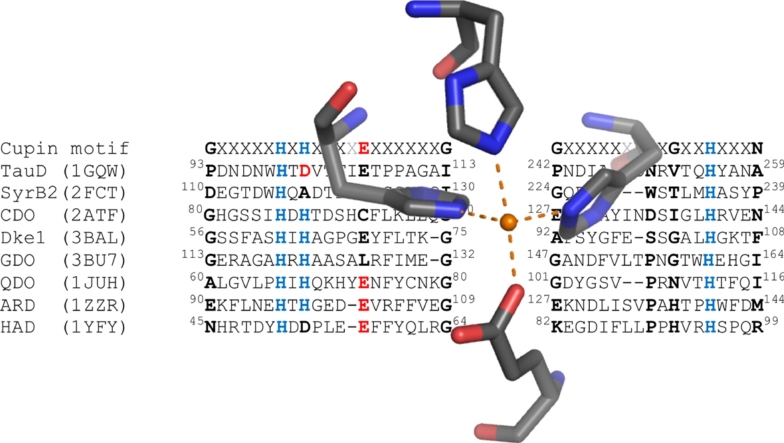

**Highlights:**

► The Cupin fold provides metal binding motif plasticity. ► The 2-His binding motif offers a platform for halogenations. ► The 3-His binding motif affects the substrate ligation mode and reactivity. ► The 3-His-1 Carboxylate Motif shows remarkable metal ion promiscuity. ► Outer shell residues impact metal center stability and catalysis.

## 1 Introduction—mononuclear non-heme-Fe(II) and O_2_ dependent enzymes (MNHEs)^1^

**Fig. 1:**
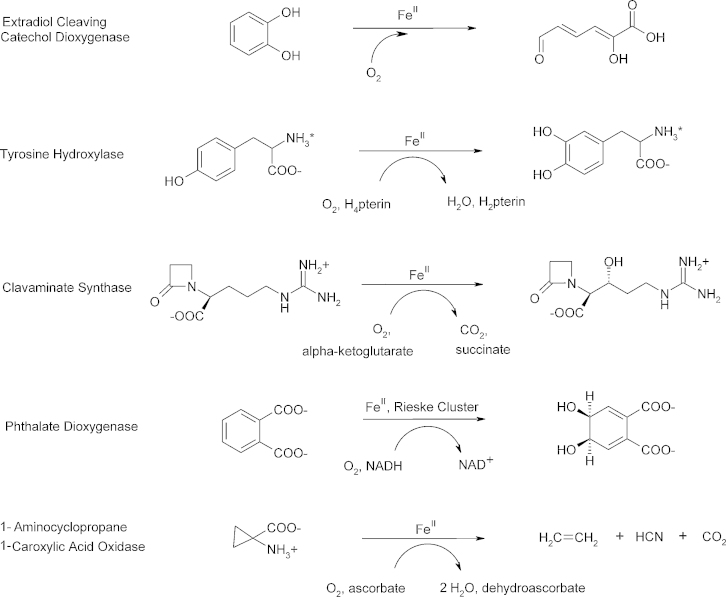
Prototypical reactions of MNHEs that show the 2-His-1-carboxylate ‘facial triad’ Fe(II)-binding motif.

MNHEs are key-players in O_2_ metabolism, on which aerobic life depends. The catalytic diversity of MNHEs, which comprises chemically challenging reactions such as hydroxylation, desaturation, C—C bond breakage and ring closure, parallels and complements the versatility of the heme containing P450 enzymes [1], [2]. MNHEs generally bind and activate O_2_ directly at the Fe(II) center and the resulting iron oxygen intermediates react with the organic substrate(s) to form the products. As the one-electron reduction of O_2_ at the iron center is thermodynamically unfavorable [3], additional electron sources are generally required to promote dioxygen activation [4]. MNHEs can be grouped according to the respective electron-source into ‘self sufficient’ cofactor independent enzymes and O_2_ activating enzymes that depend on cofactors such as pterin, α-ketoglutarate (α-KG), ascorbate or an electron providing Rieske cluster [1], [2], [5]. Examples of typical reactions that are catalyzed by MNHEs are summarized in Fig. 1.

**Fig. 2:**
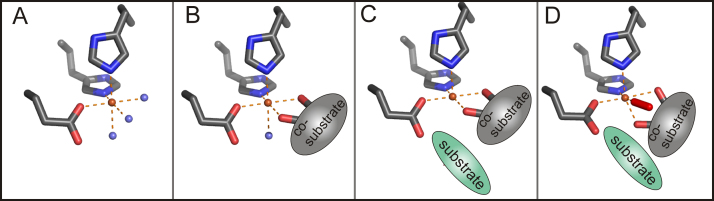
Mechanistic paradigm for dioxygen activation at the 2-His-1-carboxylate facial triad MNHEs as proposed by Solomon [1], [9] as exemplified by the active site of deacetoxycephalosporin C synthase (PDB: 1RXF).

MNHEs generally share a common metal binding motif, termed the 2-His-1-carboxylate ‘facial’ triad, where two histidine and one carboxylate-containing side chain are arranged at one face of an octahedron [6], [7], [8]. The other three coordination positions provide sites for O_2_ and the organic substrate(s) or – in the enzyme's resting state – water ligands. This general arrangement is believed to allow for an efficient coupling of dioxygen activation and substrate oxidation with water ligands protecting the metal center from auto-oxidation and uncoupled cofactor oxidation (Fig. 2 A). Only when all required co-substrates (B) and substrates are bound to the active site is a coordination site vacated, due to steric interactions and/or donor-effects of the chelating (co)substrate, (C) and O_2_ can bind to Fe(II) (D) [9], [2].

The Figure is adapted from Costas et al. [2].

## 2 Atypically coordinated mononuclear non-heme-Fe(II) centers

**Fig. 3:**
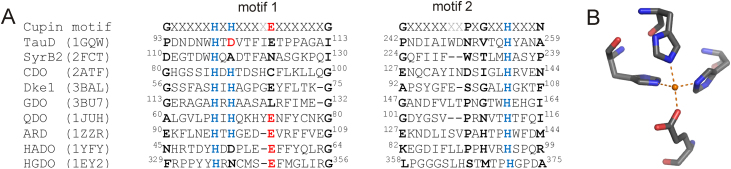
Conserved metal binding motif in cupins. (a.) An alignment of O_2_ dependent cupins that use Fe(II) as a cofactor is displayed. Metal binding residues are in blue (His) and red (carboxylate), moieties in ‘conserved’ positions are in bold. Variable residues in the cupin motif are in light grey. Abbreviations indicate the MNHEs taurine hydroxylase (TauD), SyrB2, cysteine dioxygenase (CDO), diketone dioxygenase (Dke1), gentisate dioxygenase (GDO), quercetin 2,3-dioxygenase (QDO), aci-reductone dioxygenase (ARD), 3-hydroxyanthranilate3,4-dioxygenase (HADO) and homogentisate dioxygenase (HGDO). The respective protein data bank (PDB) numbers are given in brackets. (b.) The prototypical metal center organization of a cupin-metal center is shown. (For interpretation of the references to color in this figure legend, the reader is referred to the web version of the article.)

Rather recently, a handful of O_2_ dependent MNHEs with distinct reactivities and metal binding motifs have been reported, raising the questions of why most MNHEs have the facial triad and what function is associated with the unconventional metal centers. A deeper understanding of the structural role of the metal center in MNHEs is important to fully exploit the synthetic potential of the MNHE catalytic center in the design of new functions. ‘Atypical’ non-heme-Fe(II) binding sites coordinate the metal center by (i) two histidine-, (ii) three histidine-, (iii) three histidine- and one carboxylate- and (iv) four histidine-side chains. Interestingly, with the exception of the 4-His binding motif, the atypically coordinated non-heme-Fe(II) centers are generally found in proteins that show a cupin-(β-barrel) fold. The prototypical cupin metal binding motif [10] is shown in Fig. 3, along with typically and atypically coordinated MNHEs that show a cupin fold.

In the following we summarize work done on the mechanisms of enzymes with atypically coordinated non-heme-Fe(II) centers and discuss how these structural variations impact catalysis in relation to facial triad MNHEs.

### 2.1 The 2-His motif–mononuclear non-heme-Fe(II) halogenases

#### 2.1.1 Physiological function

Mononuclear non-heme iron and α-KG dependent halogenases were first reported from the laboratory of Walsh [11], [12]. Others have been characterized since in vitro [12], [13], [14], [11], [15], [16], [17] and more have been identified by correlation of natural product structure and genetic and bio-informatic analyses [18], [19]. All of these enzymes studied to date are found in biosynthetic pathways of nonribosomal peptides (NRPs), natural metabolites with often remarkable bio-activities. The enzymes are part of an assembly line and halogenate NRP building blocks, e.g. amino acids [11], fatty acids [18] and even piperazine [15], which are then incorporated into the NRP structure. Halogenation by a 2-His coordinated Fe(II) center is also employed in order to synthesize cyclopropyl-containing amino-acids in NRP biosynthesis, where a non-oxidative, C-C bond formation and concomitant expulsion of the Cl^−^ moiety by a flavin-cofactor containing enzyme or MNHE have been reported to complete cyclopropane formation [12], [17].

#### 2.1.2 General mechanism–the role of the 2-His binding motif

**Fig. 4:**
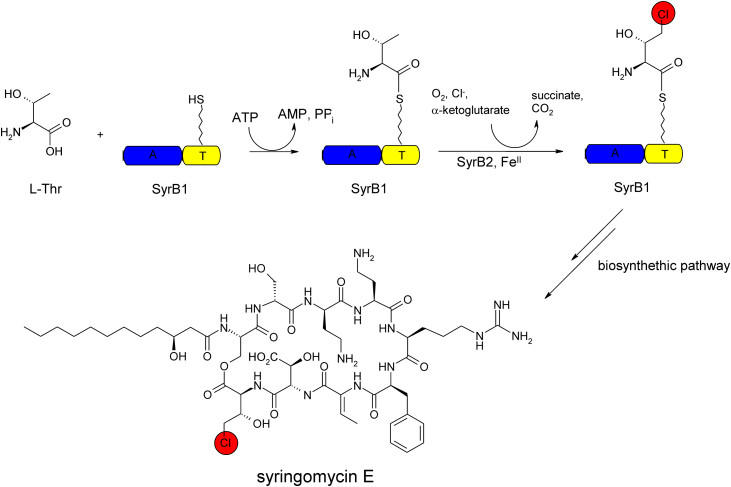
Schematic representation of biosynthesis of a halogenated threonyl-moiety in the biosynthetic pathway of the NRP syringomycin E. The threonyl moiety is tethered to the peptidyl-carrier protein domain (T) in the two-domain protein SyrB1 via a phosphopantetheinly arm. Holo-SyrB1 is then used as a substrate for the halogenase SyrB2.

Adapted from Vaillancourt [20].

The enzymes characterized so far do not act on free substrates, but on substrate moieties which are covalently linked to a carrier-protein via a phosphopantetheinyl arm. Fig. 4 depicts the halogenations of a coupled threonine by the Fe(II) and α-KG dependent halogenase SyrB2 in the biosynthetic pathway of syringomycin E.

**Fig. 5:**
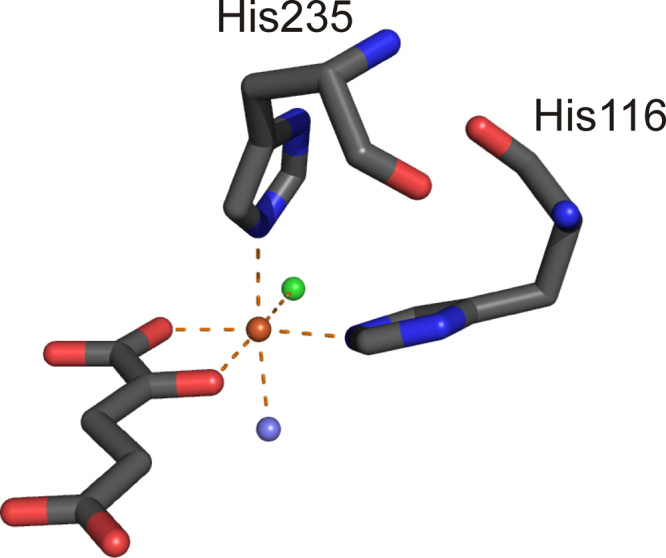
Metal binding center of SyrB2 in the presence of chloride and α-KG (PDB: 2FCT) showing six-coordinate geometry. Fe(II) is shown in orange, the chloride anion is depicted in green, water molecules are in slate blue. Oxygen and nitrogen atoms are shown in red and blue. (For interpretation of the references to color in this figure legend, the reader is referred to the web version of the article.)

**Fig. 6:**
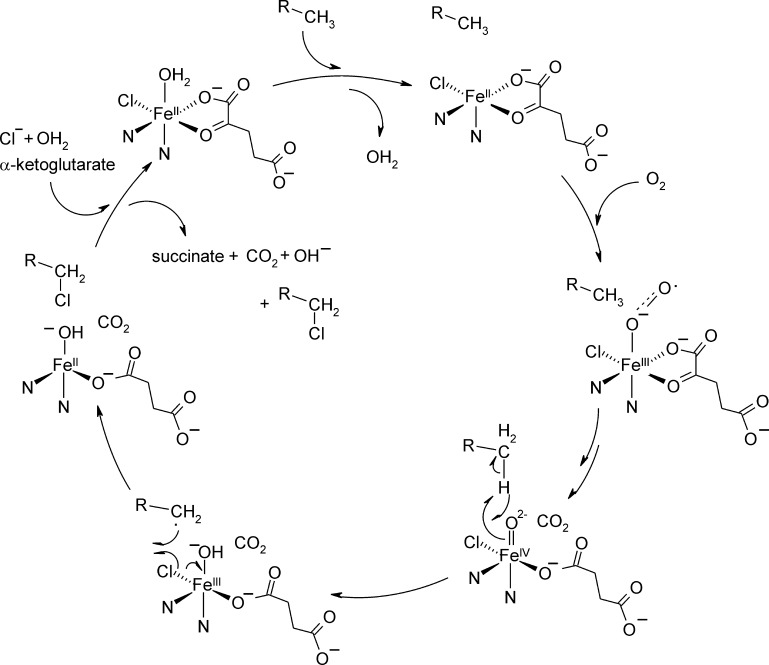
The principle reaction mechanism of non-heme-Fe(II) dependent halogenations as proposed in analogy to the mechanisms of α-KG dependent dioxygenases [21].

Sequence similarity and cofactor dependence have suggested early on that the α-KG dependent halogenases follow a similar mechanism as α-KG dependent dioxygenases, where the oxygenative decarboxylation of the cofactor and subsequent homolytic O—O cleavage yield an Fe(IV)=O intermediate. This highly reactive electrophilic agent will then abstract a hydrogen atom from the substrate, and the resulting radical will react with a metal bound halide ion [11]. It was unclear how chloride could coordinate to the metal ion subsequent to dioxygen activation and formation of a very reactive Fe(IV)=O species, however. The crystal structure of the halogenase SyrB2 that was solved by Blasiak and co-workers showed how that dilemma is solved. The structure revealed a deviation from the prototypical ‘facial triad’, where a Cl^−^ binding pocket is provided in place of the carboxylate moiety, resulting in a 2-His-Fe(II) site for metal coordination [21]. The Fe(II) containing protein structure was crystallized in the presence of α-KG. So far no metal containing crystal structure of a halogenase has been solved in the absence of α-KG and it has been suggested that Fe(II) is labile in the absence of the cofactor. Fig. 5 shows the active site of the halogenase SyrB2. The proposed chemical mechanism of the halogenating 2-His metal center is depicted in Fig. 6.

#### 2.1.3 Metal-center and active-site geometry

Magnetic circular dichroism (MCD) spectroscopy on CytC3, which catalyzes the double chlorination of L-2-aminobutyric acid, reveals that the 2-His-Fe(II) binding site shows weak affinity for Fe(II) compared to the facial triad with an additional carboxylate; The metal will only bind in the presence of α-KG as an additional ligand [22]. The Fe(II) center of CytC3 in the presence of Cl^−^ and α-KG is distorted six coordinate, however contrary to the facial triad enzyme clavaminate synthase (CS2) the 6th ligand, a water molecule, is only weakly bound. It has been argued that the carboxylate residue in the facial triad serves to stabilize the water ligand [23] as found in the crystal structure of resting CS2 [24]. The role of the carboxylate, however, does not appear to be a general feature of facial triad enzymes. Taurine hydroxylase (TauD) also shows a weak water ligand in complex with α-KG [22] and the crystal structure displays the carboxylate moiety in an orientation where the non-coordinating oxygen of the carboxylate ligand is flipped down and away from the open (i.e. water) coordination site and will therefore not partake in the stabilization of the water ligand [25], [26]. Apparently, other structural features play an additional role in maintaining hexa-coordination and thus prevent uncoupled reactivity. It has been suggested based on CS2 and TauD crystal structures that the strong versus weak water binding correlates with the closeness of bound substrate to the metal center and, consequently, to how efficiently the substrate can displace the 6th ligand in order to make the center reactive for O_2_. No structural data for halogenases with substrate bound are available but a significant number of 6-coordinate species are found via MCD spectroscopy suggesting that the bound substrate does not efficiently destabilize hexacoordination [22]. Thus the weak 6th ligand in CytC3 may serve the purpose to facilitate formation of the 5-coordinate center, in analogy to the mechanism of O_2_ activation in facial triad enzymes (Fig. 2).

The crystal structure of CytC3 has been solved in its open form where it shows low affinity for α-KG (50%) and no chloride is present n the crystal structure despite its presence during crystallization. This is in contrast to the crystal structure of SyrB2 where it has been suggested that a hydrophobic pocket and H-bonding together stabilize Cl^−^ binding in the active complex [27]. Crystal structures of the halogenase domain of CurA, which catalyzes the cryptic chlorination leading to cyclopropane ring formation in the synthesis of the natural product curacin A, have been solved in open and closed forms. A switch from the open to the closed mode apparently occurs upon α-KG binding [28]. These observations imply that major structural rearrangements are required for the halogenase metal center in order to adopt its fully active form.

Matthews and coworkers have determined that the native substrate of SyrB2 accelerates the reaction with O_2_ 8000-fold, compared to the phosphopantetheinlyated substrate carrier protein without covalently linked threonine. It was also demonstrated that chloroferryl formation in SyrB2 is 5–24-fold less efficient when other amino-acids are tethered to the substrate carrier protein SyrB1. With the alternative carrier protein CytC2, threonyl conversion was 40-fold slower compared to SyrB1, but the reactivity depended on the concentration and therefore, this effect is probably at least in part due to a lower affinity of the carrier protein to the halogenase [29]. However, reactivity with O_2_ could be induced in principle. As for alternative substrates, it was found that stable chloro-ferryl species could be generated with substrates that lacked a hydrogen atom at an abstractable, C-4, position. The resulting stable chloroferryl species was characterized via extended X-ray absorption fine structure (EXAFS) spectroscopy. Data analyses resulted in a best fit for the Fe(IV) center that included 3 shells of scatters, namely one Cl^−^ ligand at 2.31 Å, a short O/N ligand at 1.64 Å and three O/N ligands at 2.12 Å. These results were in agreement with density functional theory (DFT) calculations [29].

#### 2.1.4 Hydroxylation versus halogenation in mononulcear non-heme-Fe(II) dependent halogenases

Given that no measurable amounts of hydroxylated products have been detected in halogenase reactions [11], [21] an intriguing question was, how do the enzymes ensure halogenation versus hydroxylation in the rebound step of the mechanism (Fig. 6, last step of the reaction cycle). Five major hypotheses have been proposed: (i) Halogen-radical rebound is considered the favored route based on reduction potentials [30]. This suggestion was backed by studies of a selectively chlorinating inorganic complex [31]. In one study computational analyses using DFT calculations on the first shell residues of the halogenase active site have also shown that halogenation is energetically more favorable than hydroxylation [32]. In contrast Pandian and co-workers find comparable energies, but suggest that outer shell residues and a change in the protonation state may lead to a preference for halogenation [33]. However, mutational analysis of SyrB2 demonstrated that a reconstitution of the facial triad by mutation of the alanine residue to aspartate or glutamate did not yield hydroxylase activity, but halogenase activity was abolished [21]. This result refutes the idea that redox-potential is the overriding reason for preferred halogenation. (ii) Based on two rapidly inter-converting ferryl-species detected via Mössbauer spectroscopy [30] analogous dynamics regarding the Cl–Fe–OH rebound species have been suggested to bring about preferred halogen radical rebound. Notably only a single ferryl-species is observed in hydroxylases [34], [35], [36], [37], [38]. Other suggestions based on computational studies have been (iii) that protonation of the metal cofactor bound OH^−^ may occur, thus impeding hydroxyl rebound [33]. (iv) Alternatively, it has been proposed that CO_2_, which evolves from the α-KG cofactor after decarboxylation, may carry away the OH^−^ species as a bicarbonate so that only the halogen species is left for rebound [39]. (v) Based on the halogenase crystal structure it has been argued that substrate positioning determines the preference for halogenations [21]. Matthews and coworkers (vide supra) could show that chloroferryl formation in SyrB2 dependent on the substrate structure with some substrates actually giving accelerated rates of Fe(IV)=O decay compared to the native substrate and it was suggested that this may correlate with substrate vicinity [29]. A subsequent study used fast kinetic and product analyses on a range of deuterated and non-deuterated substrate analogues. Norvaline, which has a chain length increased by one carbon atom compared to threonine, exhibited an up to 130-fold faster ferryl-decay. This acceleration apparently correlates with a target C—H bond in the norvaline structure that comes closer to the H-abstracting ferryl species than the analogous C—H bond in threonine [40]. Intriguingly the faster H-abstraction rate came at the expense of an increase in hydroxylation products. These observations imply that halogenases position the target sites away from the H-abstracting Fe(IV)=O species in order to prevent competing hydroxylation, and this comes at the cost of lower H-abstraction rates by the ferryl-species. Summarizing, these results strongly suggest that the overriding reason for halogenations in SyrB2 is substrate positioning.

#### 2.1.5 Biomimetic complexes

**Fig. 7:**
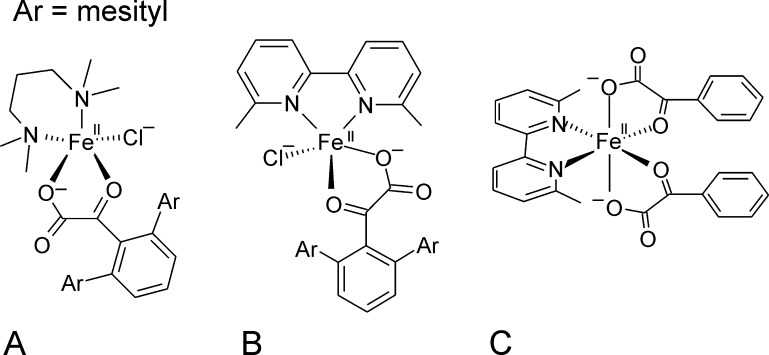
Bioinorganic complexes of Fe(II) with chloride, the bulky α-keto-acid 2,6-dimesitylbenzoyl formate and (A) N,N,N′,N′-tetramethylpropylendiamine or (B) 6,6′-dimethyl-2,2′-bipyridine mimick the active site geometry of cofactor bound Fe(II) dependent halogenases but are inert towards O_2_. (C) A complex, where unsubstituted benzoyl formate is used and the chloride ion cannot be stabilized at the Fe(II) center shows α-ketoacid decarboxylation in the presence of O_2_.

Friese and coworkers synthesized biomimetic complexes that model α-ketoglutarate coordinated halogenase active sites using bulky α-keto-acids as ligands. Two of these complexes were crystallized, which show a pentacoordinate Fe(II) center. Two coordination sites are occupied by nitrogen atoms of the respective chelate ligand, namely N,N,N′,N′-tetramethylpropylendiamine (Fig. 7A) or 6,6′-dimethyl-2,2′-bipyridine (Fig. 7B). The other coordination sites are occupied by the oxygen atoms of the bidentately coordinated α-keto-acid 2,6-dimesitylbenzoyl formate and a chloride atom. The metal center geometries are distorted square pyramidal. Over all the complexes showed a geometry that mimicked the halogenase active site quite well. However, bulky substituents at the α-keto-acid were required in order to stabilize the iron-chloride bond. A 6,6′-dimethyl-2,2′-bipyridine complex that was synthesized using the unsubstituted benzoyl formate ligand led to an octahedral complex of the chelate ligand and two bidentately bound α-keto-acids (Fig. 7C) and no coordinated chloride ion. While this complex readily decarboxylated in the presence of O_2_, the structural halogenase mimics were inert. It was argued, that the steric bulk in the complexes lead to an encapsulation of the α-ketocarboxylate group which became inaccessible to attack by a putative reactive dioxygen species. These results demonstrate how carefully halogenases need to tune their metal center structures, in order to be catalytically competent [41].

### 2.2 The 3-His motif

#### 2.2.1 Cysteine dioxygenase (CDO) and thiol oxygenating enzymes

##### 2.2.1.1 Physiological role

CDO catalyzes the first committed step in cysteine catabolism in mammals, where it oxygenates cysteine to cysteine sulfinic acid, which can then be decarboxylated to hypotaurine or transaminated to 3-sulfinylpyruvate that is spontaneously further converted to pyruvate and sulfite [42], [43], [44]. Four different CDOs from *Bacillus* and *Streptomyces* strains have been identified based on bio-informatic searches and their competence to oxidize cysteine with similar catalytic activity as rat CDO has been demonstrated [45]. Bacterial CDOs show low overall similarity with eukaryotic CDO (12–21% identity with rat CDO) but have a conserved 3-His metal binding site, according to sequence alignments.

Current data and sequence alignments suggest that the 3-His-Fe(II) center found in CDO is a common feature of thiol-dioxygenases [46], of which two more are known so far: (i) cysteamine dioxygenase (ADO) converts cysteamine – a compound which is released during coenzyme A degradation – to hypotaurine in a reaction that is analogous to CDO catalysis. CDO and ADO have important metabolic roles in mammalian metabolism. Together, CDO and ADO remove thiol compounds, produce taurine and hypotaurine and produce inorganic sulfur. ADO was identified based on bio-informatic genome analyses. Two open reading frames in the mouse genome show rather low similarity to the CDO sequence but have the key cupin metal binding motif rather well conserved. Biochemical analyses revealed that the two hypothetical paralogs of CDO indeed accept cystamine but not cysteine as a substrate [45]. (ii) Bruland and coworkers have recently identified two bacterial homologues of CDO from *Variovorax paradoxus* TBEA6 and *Ralstonia eutropha* H16. These enzymes catalyze the oxidation of 3-mercaptopropionate to sulfinopropionate but do not convert cysteine and cysteamine [47]. Both enzymes show strong similarity to bacterial CDOs, based on the motif defined by Dominy and co-workers [45].

##### 2.2.1.2 Metal-center and active-site geometry

Electron paramagnetic resonance (EPR) spectroscopic studies have shown that the affinity of the Fe(II) center to the O_2_ mimic NO is strongly increased in the presence of substrate [48] demonstrating the ‘activation’ of the metal center for O_2_ reduction by substrate coordination. It has been suggested that bidentate coordination of the substrate lowers the reduction potential of Fe(II), thus priming it for the reduction of dioxygen. Also, the presence of a water ligand in the resting enzyme may protect Fe(II) from oxidation. Notably, the substrate analogues cysteamine, 3-mercaptopropionate and 1-mercaptopropane did not achieve a significant increase in affinity for NO, possibly due to low substrate affinity to the active site.

A low spin {FeNO}^7^ (S = 1/2) species resulted, contrary to the typically observed high spin {FeNO}^7^ (S = 3/2) complexes observed in 2-His-1-carboxylate enzymes. It was argued that this electronic difference may be caused by the amido-rich coordination sphere compared to 2-His-1-carboxylate enzymes. While results from EXAFS spectroscopy suggested that coordination of cysteine via its carboxy-moiety was the most likely mode of coordination [49], MCD spectroscopy of the cysteine-coordinated complexes revealed near UV transition bands, which were red-shifted for the seleno-cysteine-bound analogue, supporting direct coordination of the thiol-group to the Fe(II) cofactor. MCD data did not define the geometry of the resting active site of CDO. A visible transition of the cysteine-coordinated Fe(III)-CDO further indicated direct coordination of cysteine to the metal cofactor [50].

**Fig. 8:**
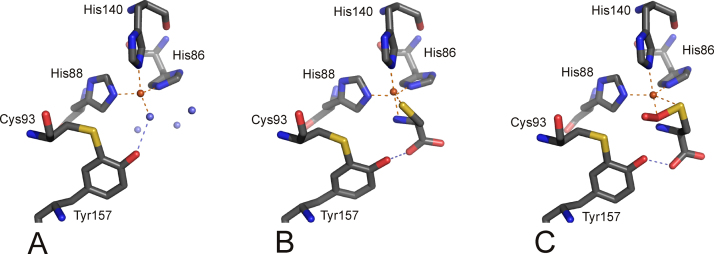
Active site of (A) resting CDO (PDB:2B5H), (B) L-cysteine bound CDO (PDB:2IC1) [61] and (C) persulfenate coordinated CDO (PDB:3ELN) [51]. Metal binding amino acids and the covalently linked outer sphere residues Cys93 and Tyr157 are shown. Note that the resting CDO structure contains an Fe(III) ion, while substrate and intermediate bound structures shown have an Fe(II) as active site metal ion. The iron ion is in orange, water molecules are slate blue; oxygen, nitrogen and sulfur-atoms are depicted in red, blue and yellow, respectively. H-bonds are indicated as slate blue dashed lines. (For interpretation of the references to color in this figure legend, the reader is referred to the web version of the article.)

Crystal structures of free eukaryotic CDOs from mouse [51] and rat [52] have been solved and show a 3-His metal binding center (Fig. 8A). Depending on the bound metal ion, Ni(II) [51] or Fe(III) [52], one or three additional water ligands were found in the active site, giving tetrahedral or octahedral coordination, respectively, whereby the localization of the actual ligands did not significantly change. A structure of human CDO which was anaerobically co-crystallized with cysteine shows that cysteine [53] is coordinated to the Fe(II) center via its sulfur and amino group supporting the mode of substrate coordination as initially proposed by McCoy and coworkers (Fig. 8B) [52]. Remarkably, the crystal structures of eukaryotic CDO revealed that cysteine Cys93 is covalently linked to active site tyrosine Tyr157 (using rat numbering; Fig. 8). This cysteine is prototypical for eukaryotic CDOs and replaces the glutamate residue that is generally found in the cupin-motif.

##### 2.2.1.3 The role of the tyrosine-cysteine crosslink in the active site

The thioether Cys-Tyr crosslink, which is found throughout eukaryotic CDO crystal structures has raised the question of a putative role as a ‘radical cofactor’ in the catalytic mechanism of cysteine oxygenation. Such cofactors have been described for galactose oxidase [54], glyoxal oxidase [55], [56] and a sulfite reductase [57]. Both forms, CDO with and without the Cys-Tyr crosslink, are found in tissues and cell cultures [43], [58], [59], [60]. A radical scavenger could not significantly reduce catalytic activity of CDO at a 1000-fold excess [47], supporting a scenario where catalysis does not involve a mechanism that depends on a putative tyrosine radical as, e.g. described in galactose oxidase.

It has been demonstrated that the crosslink forms under turnover conditions, i.e. in the presence of cysteine, Fe(II) and O_2_, and it promotes enzyme activity [58]. Notably, the cross-link forms slowly, after an estimated 800 turnovers. Enzymatic activity in the absence of a crosslink is markedly reduced [43], [60]. Mutational analyses give a two- and five-fold decrease in activity for the substitution of Cys93 by serine and a ten- and twenty-fold lower activity regarding the Tyr157 to phenylalanine substitution in rat and human CDO, respectively [58], [61]. Furthermore, the cross-link apparently enhances catalytic stability about two-fold [58]. Ye and co-workers have found that a reduced catalytic activity correlates with lower iron content in the CDO variants and have suggested that this is the actual reason for activity loss in the variants. A lowered affinity for the Fe(II) cofactor may impact apparent turnover numbers in the presence of Fe(II) in the assay, as described by Dominy [58]. A further hypothetical role, proposed by Ye and co-workers, is that Tyr157 may stabilize radical intermediates during catalysis and thus prevents the uncoupling of the reaction. Taken together, data support a scenario where the Tyr-Cys crosslink stabilizes the resting iron center and possibly reaction intermediates (vide infra).

Notably, bacterial CDOs do not only show low over-all similarity with eukaryotic CDOs (12–21% identity with rat CDO), but many of them also do not have the conserved cysteine, which forms the thioether bond to the tyrosine in the active site of eukaryotic structures. This, along with he fact that mutants are active and the enzyme turns over 800 times before the crosslink is formed, suggests that the thioether bond is not a crucial structural requirement for cysteine oxygenation. The structure of a bacterial CDO has been deposited in the Protein Data Bank (PDB). The metal center shows octahedral geometry, and a comparison with eukaryotic structures suggests that the Tyr-Cys crosslink in eukaryotic CDOs may lead to a distortion of the water-shell geometry in eukaryotic CDOs [61]. The bacterial structure so far remains unpublished (PDB: 2GM6).

##### 2.2.1.4 Reaction mechanism

**Fig. 9:**
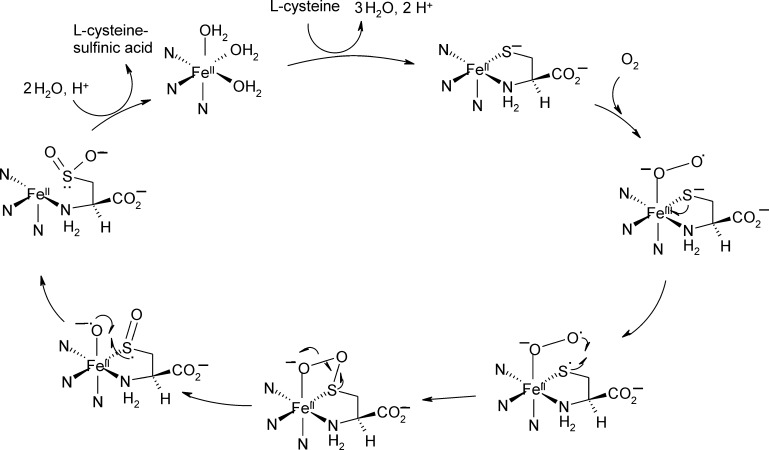
Reaction mechanism of cysteine oxidation as proposed based on the mouse crystal structure, adapted from ref. [52].

Several reaction mechanisms have been proposed for CDOs. Here, based on accumulated evidence that the substrate's sulfur atom is coordinated to the metal ion during catalysis, three principle mechanisms are discussed that follow this scenario. The mechanism that was initially proposed based on the crystal structure of resting Ni(II)-CDO (Fig. 8A) is shown in Fig. 9. It proceeds via substrate binding and subsequent activation of O_2_ at the iron center, and – after oxidation of the sulfur moiety by Fe(III) – an attack of the activated oxygen species at the sulfur. The resulting four-membered ring then collapses, thus transferring one oxygen onto the sulfur atom. Finally the second, metal-bound oxygen species is also transferred to the sulfur moiety.

**Fig. 10:**
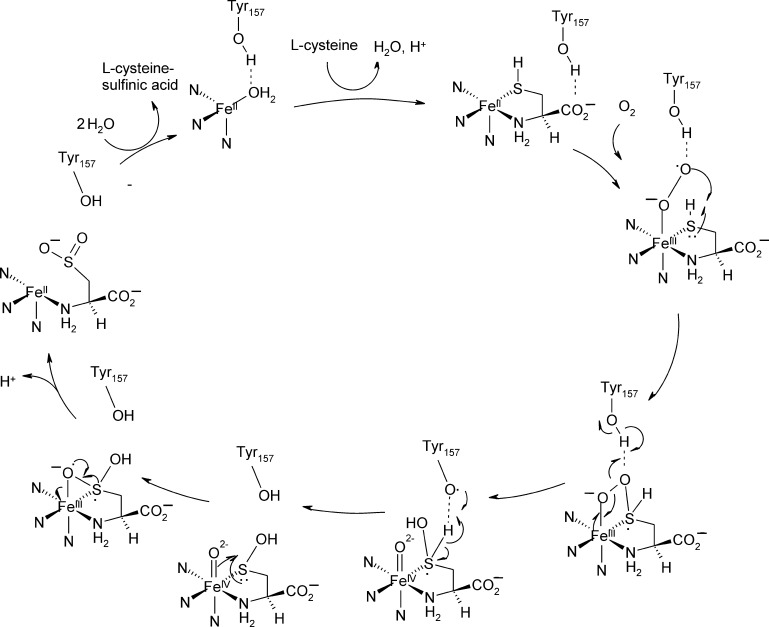
Reaction mechanism of cysteine oxidation as proposed based on the human, substrate bound crystal structure, adapted from ref. [61].

An alternative mechanism has been suggested by Ye and co-workers based on the structure of human Fe(II)-CDO in complex with substrate (Fig. 8B) [61], where a role of the thioether bonded Tyr157 residue has been proposed. As described in Fig. 10, cysteine coordinates to the tetrahedrally coordinated active site as found in the structure of resting Fe(III)-CDO from rat [53]. The resulting five-coordinate center binds and activates O_2_, which then attacks the iron-bound sulfur. The resulting four membered ring cleaves homolytically at its O—O bond, resulting in a high-valent Fe(IV)-oxo species, which then attacks the sulfur and transfers the second oxygen moiety. It is suggested that Tyr157 stabilizes the resting metal center and the transition states of dioxygen activation and Fe(IV)-oxo species formation.

**Fig. 11:**
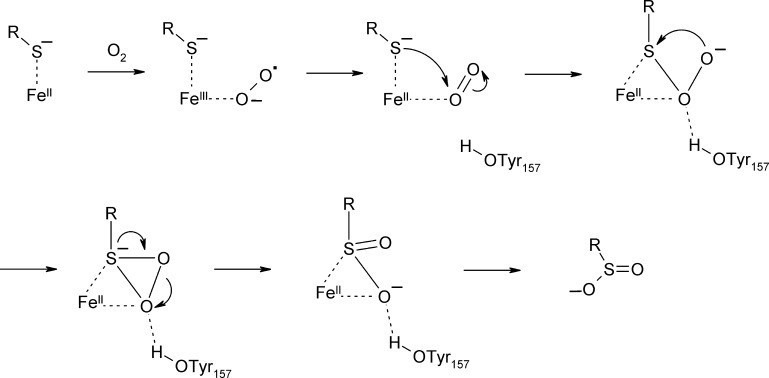
Reaction mechanism of CDO involving attack of the proximal oxygen atom at the metal coordinated sulfur atom of the substrate, as proposed based on a crystal-structure which has a persulfenate bound to the Fe(II) center.

Adapted from ref. [51].

A structure of CDO with a putative persulfenate intermediate coordinated to the Fe(II) cofactor has been solved by Simmons and coworkers (Fig. 8C), [51]. Based on the active site organization of this structure it has been suggested that not the iron-terminal but the iron-proximal oxygen atom of the proposed superoxide intermediate oxidizes the metal bound sulfur atom (Fig. 11).

Very recently, Crawford and coworkers [62] have described the catalytic turnover of cysteine by Fe(III)-CDO and superoxide. Catalytic turnover rates in this chemical rescue experiment were at least 200-fold slower than in wild type enzyme and it is therefore still unclear how analogous this reaction is to that of the native enzyme. The fact that this conversion is feasible in principle, supports a mechanism that proceeds via an Fe(III)-superoxo intermediate.

*Computational analysis:* Computational analyses of the reaction mechanism via QM and QM/MM calculations suggest a Fe(IV) intermediate in the reaction cycle [63], [64] similar to the mechanism proposed by Ye and co-workers [61]. Notably, QM/MM calculations find the oxygenation of sulfur by the Fe-proximal oxygen, as has been proposed based on the persulfenate containing crystal structure, to be a high energy pathway. It has been suggested that the intermediate found in the crystal structure may actually reflect a side product of the reaction [63].

##### 2.2.1.5 Biomimetic complexes

**Fig. 12:**
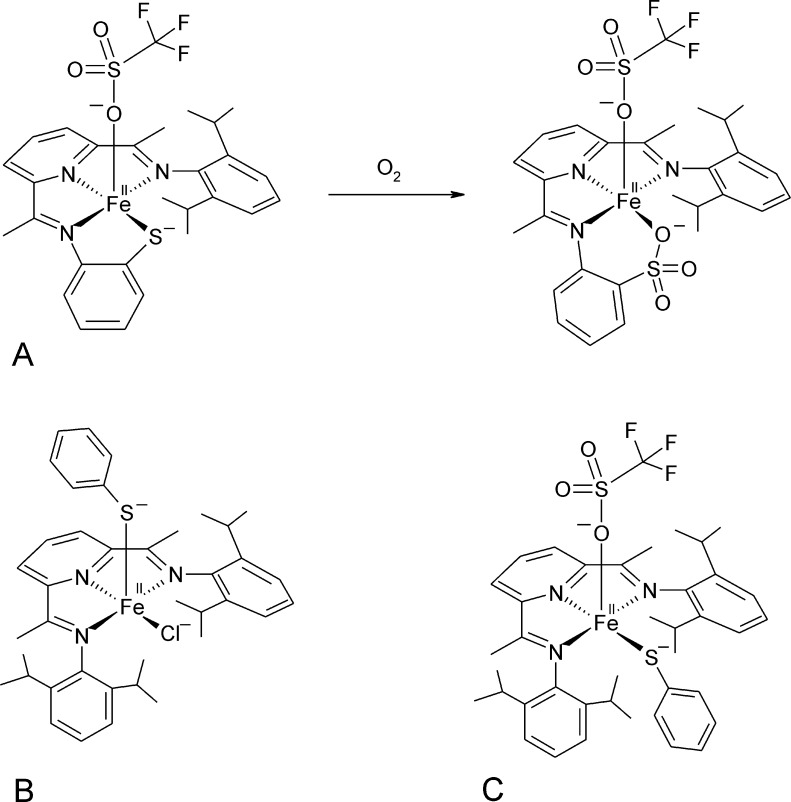
Biomimetic complexes with the potential to oxygenate thiols. (A) The Fe(II)(LN3S)(OTf) complex (Fig. 12A, left) has the thiol group covalently linked to the chelating ligand and incubation with O_2_ leads to the sulfonate product. By analogy, (B) the non-covalently linked thiolate ligand in cis-position of complex Fe(II)(^iPr^BIP)(SPh)(OTf) is oxygenated, while (C) for the analogous complex Fe(II)(^iPr^BIP)(SPh)(Cl), which has the thiol ligand in trans position, iron oxygenation to Fe(IV)=O is observed instead.

Biomimetic complexes which oxygenate a sulfur ligand upon reaction with O_2_ [65], [66] have been synthesized recently. A bis(imino)pyridine (BIP) ligand scaffold was employed, which contained a thiolate moiety. The resulting Fe(II)(LN3S)(OTf) complex (Fig. 12A, left) showed distorted square pyramidal geometry, with the trifluormethanesulfonate (OTf^−^) ligand occupying the axial position. The complex was reactive with O_2_ and in mass spectrometric analysis a doubly oxygenated species was observed which disappeared over time. The product complex could not be crystallized, but based on product analysis a sulfonato complex was proposed as the final product (Fig. 12A, right). Based on ^18^O incorporation studies and mass spectrometry it was shown that two atoms of molecular oxygen were initially incorporated into the sulfonate product, followed by a third insertion of labeled oxygen after longer incubation times. The analogous Zn(II) complex was inactive. In a subsequent work, two complexes were synthesized, which bind a thiolate ligand that is not a part of a chelate system. Isopropyl-BIP (^iPr^BIP), phenylthiol (PhSH) and Fe(II) yielded two complexes that differed in geometry, when crystallized in the presence of Cl^−^ or OTf^−^, respectively (Fig. 12B and C). Both complexes show five coordinate Fe(II) ions. However, while (^iPr^BIP)(SPh)(Cl) has the PhS^−^ ligand in pseudoaxial position in relation to the N_3_Cl^−^ plane (Fig. 12B), the thiolate ligand is in a pseudoequatorial arrangement in (^iPr^BIP)(SPh)(OTf) (Fig. 12C). Notably, only the latter complex, which has the thiolate ligand in cis-position to the open coordination site of Fe(II) afforded sulfur oxygenation in the presence of O_2_ [65]. (^iPr^BIP)(SPh)(Cl), which has the thiolate in trans position, by contrast formed an Fe(IV)=O species andPhS^−^ was concomitantly oxidized to the disulfide. It has also been shown that a thiolate-ligand is crucial for the activation of the non-heme bis(imino)pyridine complex by lowering the reduction potential. These observations further support a mechanism that proceeds via direct coordination of sulfur to the metal center and where the relative positioning of the thiol moiety plays a critical role in determining, whether oxygenation occurs at the iron or sulfur.

#### 2.2.2 Diketone dioxygenase (Dke1)

##### 2.2.2.1 Physiological role

Dke1 was the first committed enzyme of the acetylacetone (acac) degradation pathway in *Acinetobacter johnsonii* (DSMZ-ID: 98–849).^2^ The organism was isolated from sewage based on its ability to grow on acac as the sole source of carbon [67]. Dke1 catalyzes the O_2_-dependent cleavage of acac into methylglyoxal and acetate, which are then further metabolized. Data mining indicates that the enzyme may be widespread among microorganisms from different sources, as highly similar sequences were also isolated from the Sargasso sea during a shotgun sequencing project [68]. Furthermore, acac cleavage activity has been demonstrated from a homologous *Burkhoderia xenovorans* protein [69].

##### 2.2.2.2 Metal-center and active-site geometry

**Fig. 13:**
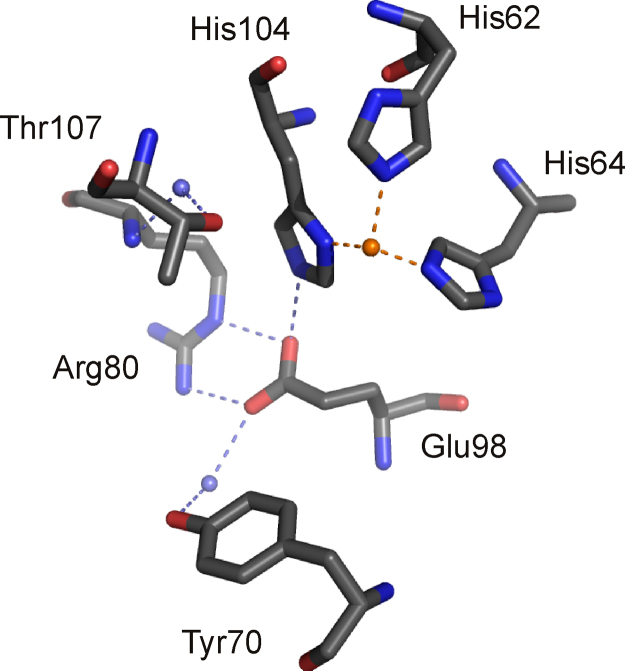
3-His metal center of Dke1 and hydrophilic active site residues, Tyr70, Arg80, Glu98 and Thr107 (PDB: 3BAL) [70]. The metal ion in the structure, which is Zn(II), is shown in orange, water molecules are in slate blue, oxygen atoms and nitrogen atoms are in red and blue, respectively. H-bonds are indicated as slate blue dashed lines. (For interpretation of the references to color in this figure legend, the reader is referred to the web version of the article.)

Dke1 is a functional homotetramer composed of subunits of 153 amino acids in length. The Dke1 structure shows a cupin fold and also displays the prototypical cupin metal binding motif. Notably, in the crystal structure (PDB: 3BAL) [70], the conserved Glu69 residue of the cupin signature is not positioned in the active site and mutational analysis confirms that Glu69 is not required for metal binding or catalysis [71]. By contrast, the substitution of the three histidine residues His62, His64 and His104, which coordinate the metal ion in the crystal structure (Fig. 13), leads to a marked decrease in Fe(II) binding-affinity and activity loss [72]. However, the crystal structure of Dke1 contains a Zn(II)-ion in the active site and very recent molecular dynamic studies suggest that the Fe(II) containing structure indeed coordinates the metal cofactor via the 3 histidines and Glu98. Upon substrate binding Glu98 is displaced and the catalytically competent complex forms a 3-His center, similar to the crystal structure [73].

Circular dichroism (CD) and MCD spectroscopic studies of the 3-His metal center of Dke1 have shown that the enzyme follows the mechanistic paradigm of O_2_ activation of the facial triad enzymes, which is outlined in Fig. 2. The resting enzyme Dke1 shows no significant difference in the 6-coordinate metal ion's ligand field compared to the facial triad enzyme CS2 and becomes a mixture of 5- and 6-coordinate species upon binding of acac. DFT calculations support loss of water driven by binding of the strong donor acac, which decreases water affinity by ∼2.8 kcal^.^mol^−1^. The binding of the organic co-substrate α-KG co-substrate dependent facial triad enzymes (e.g. CS2) in general do not lead to a loss of water and it has been argued that there, the facial triad's prototypical carboxylate ligand stabilizes the 6th water ligand via its non-coordinated oxygen. Thus organic co-substrate dependent facial triad enzymes ensure that no decoupled oxygenation of the cofactor takes place until the organic substrate is bound, which then induces a steric displacement of the 6th ligand.^3^ A comparison of Dke1 and the facial triad enzyme 4-hydroxyphenylpyrvate dioxygenase, both chelated with acac, gives a generally similar geometric and electronic structure, however the metal-to-ligand charge transfer band of Dke1 is shifted to higher energy by 1000 cm^−1^. This reflects the higher positive charge in the active site of the 3-His center of Dke1, which decreases the energy of the d manifold [74]. Combined kinetic and CD/MCD studies on the effect of pH on the Dke1 active site showed a depressed p*K*_a_ for bound water (∼8.2) at the 3-His center and that deprotonation of the water ligand negatively affects the velocity of substrate binding to the metal center. A water-hydroxide equilibrium at this pH range is not typically found for facial triad mononuclear non-heme-Fe(II) sites and may be a result of the distinct net charge of the 3-His motif compared to its 2-His 1-carboxylate counterpart [75].

##### 2.2.2.3 Reaction mechanism

**Fig. 14:**
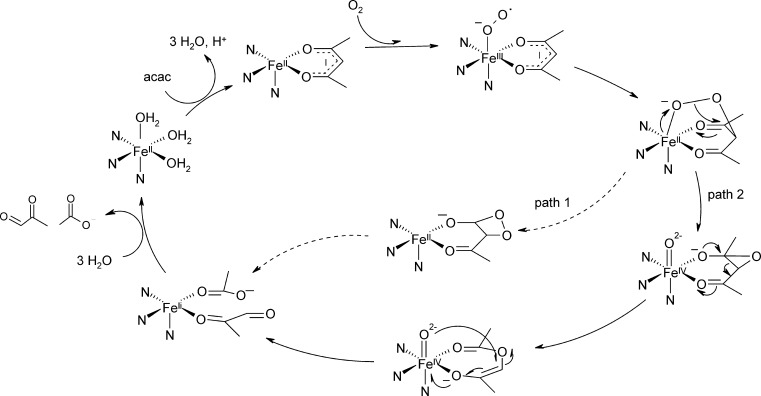
Reaction mechanism of diketone cleavage by Dke1. As the initially proposed reaction mechanism via a dioxetane intermediate (path 1, dashed arrows) [77] is high in energy, an alternative, energetically more feasible C—C bond cleavage mechanism has been proposed based on DFT calculations (path 2) [79].

Dke1 is strictly dependent on the Fe(II) cofactor, although the catalytic Fe(II) site of Dke1 can be substituted by various other metal ions such as Fe(III), Co(II), Ni(II), Cu(II), and Zn(II). Dke1 converts a wide variety of β-diketone substrates capable of forming cis-β-keto-enolate structures [76]. According to ^18^O labeling studies, one atom of molecular oxygen is incorporated into each acetylacetone cleavage product, namely acetate and methylglyoxal. Consequently Dke1 has been classified as a dioxygenase [77]. The enzymatic activity of Dke1 linearly depends on the concentration of O_2_ up to 1.2 mM, which is the limit of O_2_ solubility under ambient conditions. The rate determining step has consequently a second order rate constant $k_{\text{cat}}/K_{m{\text{O}}_{2}}$ of 35 s^−1^ mM^−1^ for acac under standard conditions. Enzyme activity varies by a factor of up to 10^5^ as a function of the electronic substrate structure and a combination of quantitative structure activity relationship analysis and single-turnover kinetics revealed that the energy of the highest occupied molecular orbital directly correlates with the rate of O_2_ dependent cleavage of the respective chromogenic diketonate-Fe(II)-Dke1 complex. Based on these observations it was concluded that O_2_ reduction is the rate determining step of the enzymatic reaction. The aromatic inhibitor 2′-hydroxyacetophenone lowers the redox potential to a degree comparable to acac in inorganic Fe(II) complexes but it is energetically prohibited from forming a C—O bond with O_2_ as this would compromise the ligand's aromaticity. Therefore, it was suggested that O_2_ reduction and C—O bond formation cannot be kinetically resolved and the rate determining step is the concerted oxidation of acac to the respective C-3 centered peroxidate intermediate by O_2_ [78]. The pattern of C—C bond scission upon diketone conversion strongly depends on electronic substituent effects in Dke1 with favorable C—C bond cleavage adjacent to the more electron withdrawing group, indicating a negatively charged transition state during C—C bond fission. Consequently a peroxidate cleavage mechanism via a dioxetane intermediate was proposed (Fig. 14, path1) [77].

*Computational analysis:* More recently the reaction mechanism was revisited using QM calculations. The formation of a C-3 centered peroxidate intermediate was substantiated and a preceding Fe(III)-O•− intermediate that was high in energy was found which rationalizes why O_2_ reduction and C—O bond formation cannot be decoupled. According to computational analysis, the O—O bond of the peroxidate intermediate cleaves yielding a Fe(IV) = O species and – as the oxygen distal to the iron attacks the dicarbonyl's C2 – an epoxide intermediate. The latter collapses into an ester compound, which is then attacked via the Fe(IV) = O species and cleaved to the final products (Fig. 14, path 2) [79]. The C—C bond cleavage step proceeds via a negatively charged intermediate and is therefore in line with the previously observed diketonate cleavage pattern [77].

##### 2.2.2.4 The impact of outer shell residues on catalysis

Hydrophilic outer shell residues have a significant impact on the functionality of the 3-His binding site, as demonstrated by mutational and enzyme kinetic analyses [75]. Substitution of Thr107, an outer shell residue which is in H-bonding distance of putative metal-bound waters, leads to Fe(II) loss, presumably by destabilizing water ligands (Fig. 13). Glu98^4^, which is also located in the 2nd shell of the metal center and hydrogen bonds with Nɛ2 of His104, on the contrary, is not required for metal cofactor binding. It stabilizes the 6-coordinate geometry of the metal center and promotes ligand-exchange, thus accelerating substrate binding. Upon its substitution the metal center of resting Dke1 becomes partially 5-coordinate, whereby, notably, auto-oxidation rates are not measurably increased. The three hydrophilic residues Glu98, Arg80 and Tyr70, which form a hydrophilic gate to the active site cavity and coordinate an outer shell water molecule (see Fig. 13), are crucial for efficient O_2_ reduction. Substitution by alanine and – in the case of Glu98 – also glutamine leads to a ∼50–100-fold decrease in O_2_ reduction rates, while the structurally conservative mutation of Tyr70 to phenylalanine leads to a 3-fold decrease of the catalytic rate. It has been proposed that the hydrophilic gate may stabilize the transition state by providing a hydrogen bond that stabilizes the negative charge on the evolving superoxide species or, alternatively, that it promotes the O_2_-reactive 5-coordinate species by limiting water access to the active site.

##### 2.2.2.5 Substrate-promiscuity of Dke1

The 3-His center of Dke1 has been probed for enzymatic activity towards substrates of other prototypical non-heme-Fe(II) enzymes [80]. Notably, low catechol extradiol-cleavage activity was observed, which demonstrates the principle potential of the 3-His center to perform a facial triad-type reaction. Also, substantial reactivity towards maltol was found and kinetically characterized. Maltol can be viewed as a truncated quercetin-substructure. Maltol cleavage was strictly Fe(II) dependent, and no reactivity was observed for Dke1 variants that had been substituted by Cu(II), Mn(II), Zn(II), Co(II) or Ni(II). Notably, phenylpyruvate and 4-hydroxyphenylpyruvate were oxygenatively cleaved at the C2-C3 bond in Dke1. In contrast, 4-hydroxyphenylpyruvate converting dioxygenases are facial triad enzymes that decarboxylate their substrate similar to the α-KG dependent dioxygenases. Substrate binding was subsequently further characterized spectroscopically via low temperature CD/MCD and resonance Raman spectroscopy and it was found that the deviating reactivity stems from a distinct binding mode. While the α-oxo-acids are generally bound in their mono-anionic form in the facial triad employing oxygenases, 4-hydroxyphenylpyruvate was coordinated in its di-anionic form, as an enolate, at the 3-His center of Dke1 [79]. DFT calculations have shown that enzymatic conversion of the di-anionic form proceeds via a Fe(III)-HPP•-O_2_^2−^ species, which rearranges to a Fe(IV)=O-epoxide and is then cleaved to benzaldehyde and oxalate, while mono-anionic binding at the 2-His-1-carboxylate center initiates the prototypical keto-acid dioxygenase mechanism.^5^ The distinct cleavage pattern at the 3-His-Fe(II) center compared to the 2-His-1-carboxylate site suggests that one role of the metal binding motif is to stabilize the correct ionization state in the metal bound (co)substrate molecule.

##### 2.2.2.6 Biomimetic complexes

**Fig. 15:**
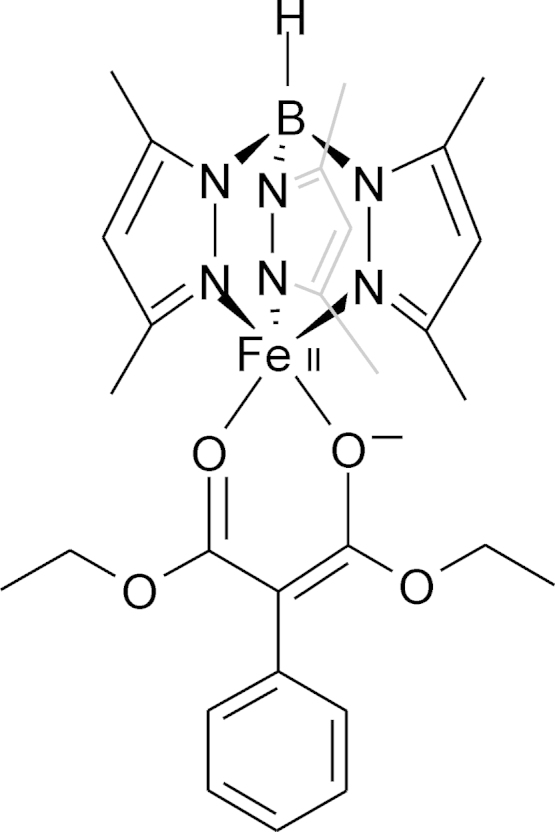
Structural representation of an Fe(II) complex mimicking β-dicarbonyl cleavage. Hydridotris(3,5-dimethylpyrazol-1-yl)borate is the chelating ligand and diethyl 3-phenylmalonate is the β-dicarbonyl ligand, which undergoes cleavage in the presence of O_2_.

A bio-mimetic Fe(II) complex was synthesized, using hydridotris(3,5-dimethylpyrazol-1-yl)borate as a chelating ligand and diethyl 3-phenylmalonate as the β-diketonate ligand and a functional model for O_2_ dependent diketone cleavage by Dke1 was obtained (Fig. 15) [82]. Notably, the native substrate of Dke1, acac, was not cleaved in an analogous complex. Incorporation of one atom of molecular oxygen into each cleavage product, ethyl benzoylformate and CO_2_ was confirmed by ^18^O labeling studies. The authors have proposed that the activated primary superoxo-species performs an attack on C-2 of acac in Dke1. This mechanism is, however, not supported by DFT calculations (vide supra). More recently, a series of high-spin iron(II)-β-diketonato complexes has been spectroscopically and electronically characterized, using a range of substituted diketonates (not shown). Substituted hydrotris(pyrazol-1-yl)borate and the neutral tris(2-phenylimidazol-4-yl)phosphine were employed as ligands and a range of five-and six coordinated Fe(II) complexes was obtained [83]. The inorganic complexes showed comparable electronic properties as the analogous complexes of Dke1 with the various substituted diketonates [78]. Studies regarding the reactivity of these biomimetic complexes towards O_2_ are not published so far.

#### 2.2.3 3-His coordinated aromatic ring-cleaving dioxygenases

**Fig. 16:**
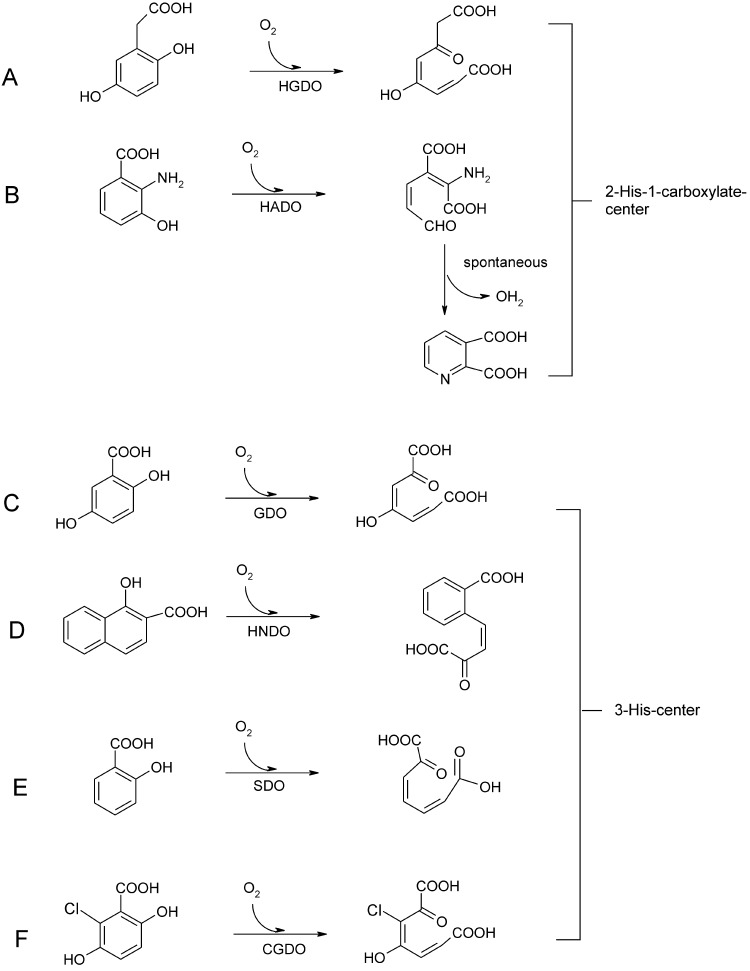
Ring cleavage reactions of the facial triad enzymes (A) homogentisate dioxygenase (HGDO) and (B) 3-hydroxyanthranilate 3,4-dioxygenase (HADO) and their 3-His coordinated counterparts (C) gentisate dioxygenase (GDO), (D) 1-hydroxy 2-naphthoate dioxygenase (HNDO), (E) salicylate 1,2-dioxygenase (SDO) and (F) 5-chloro-gentisate 1,2-dioxygenase (CGDO).

Ring-fission dioxygenases are key-enzymes for the aerobic degradation of aromatic structures. Fe(III) intradiol-cleavage catechol dioxygenases break the ring's C—C bond between the two geminal hydroxyl-groups, while their counterparts, the extradiol cleavage dioxygenases depend on Fe(II) and cleave the bond in the 2,3 position of the 1,2-catechol structure [84], [85]. The latter have the common 2-His-1-carboxylate coordinated Fe(II) center [6]. A third group of dioxygenases cleaves aromatic structures that do not contain geminal diols. These enzymes generally show a bicupin structure, i.e. the protein folds into two cupin domains [10]. Among these enzymes are the dioxygenases that convert 3-hydroxy-anthranilate [86], homogentisate [87], 2-aminophenol [88], 5-amino-salicylate [89], 5-nitro-salicylate [90], 5-chloro-salicylate [91], gentisate [92], salicylate [93], and 1-hydroxy-2-naphthoate [94]. The latter four conversions take place at an enzymatic 3-His coordinated Fe(II) center, while the first two substrates are cleaved by enzymes that display the ‘classical’ 2-His-1-carboxylate binding signature. The other enzymes are not structurally characterized. Reactions catalyzed by the third grouping of dioxygenases are shown in Fig. 16.

##### 2.2.3.1 Gentisate 1,2-dioxygenase (GDO)

**Fig. 17:**
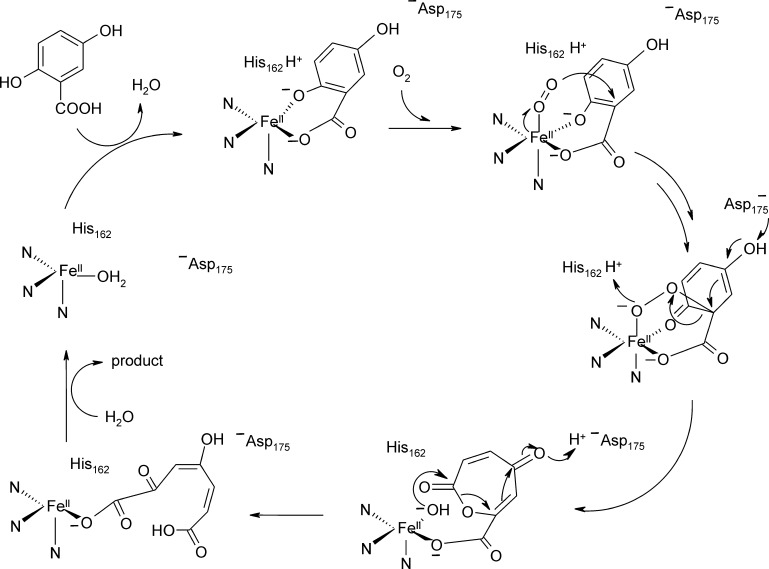
Proposed gentisate cleavage mechanism, adapted from references [97], [101]. The principle mechanism of Harpel and coworkers suggest initial activation of O_2_ by electron transfer from Fe(II), followed by attack of the activated oxygen species at C2 of the substrate, resulting in the peroxidate intermediate. The 3-His metal binding motif and metal coordinated water molecules as well as outer shell residues that assist in catalysis are inferred based on the crystal structure [100], [101].

GDO cleaves the C1—C2 bond of gentisate and a broad range of 3- and 4-substituted substrate analogues – among them alkyl- and halogen substituted structures – by concomitant incorporation of molecular oxygen [95], [96]. Biochemical studies have established that ferrous iron is the catalytically competent metal cofactor [95], [97], [98]. EPR studies of GDO complexes with the O_2_-surrogate NO showed an *S* = 3/2 species for resting enzyme and upon substrate binding the signal was significantly perturbed [97]. When performed in H_2_^17^O the signal of resting enzyme was significantly broadened, while substrate binding led to a line sharpening. It was suggested that this is due to replacement of water ligands from the active site. Furthermore, when ^17^O labeled gentisate analogues were used as ligands, signal broadening was again observed for labeled carboxylate- and 2-OH substituents, but not for the 5-OH substituent, suggesting that gentisate chelates the Fe(II) cofactor via the carboxylate and the adjacent hydroxy moieties. Consequently a reaction mechanism has been suggested where coordination of the electron donating substrate lowers the redox-potential so that Fe(II) can activate O_2_ by transfer of an electron for attack of the aromatic ring. The ring shows a positive partial charge at C2 due to charge delocalization between the substrate and metal. Subsequent heterolytic cleavage of the peroxidate intermediate and Criegee rearrangement would lead to incorporation of one atom of oxygen. Hydrolysis of the resulting ester bond leads to the final product. The mechanism is outlined in Fig. 17. In this mechanism the redox-active metal acts as an electron conduit. Note that more recent quantum-mechanic studies have shed further light on the reaction pathway (vide infra and Fig. 20) [99].

**Fig. 18:**
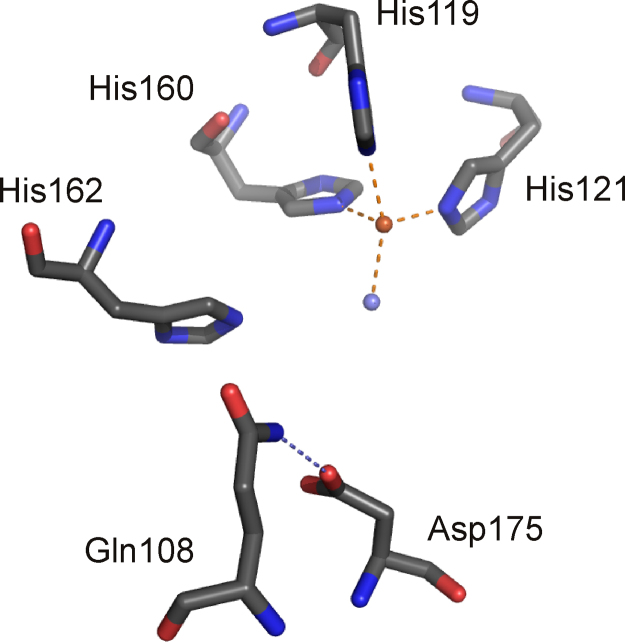
Iron-center of GDO from *S. pomeroy* (PDB: 3BU7) in the N-terminal domain of the bicupin structure. The metal ion is coordinated by the metal binding residues His119, His121 and His160. Outer-shell residues Gln108, His162 and Asp175, which have been suggested to partake in acid–base catalysis are also shown. Note that the metal ion is presumably present in its ferric form in the crystal structure. Oxygen atoms and nitrogen atoms are shown in red and blue, respectively. Water molecules are shown in slate blue. H-bonds are indicated as slate blue dashed lines. (For interpretation of the references to color in this figure legend, the reader is referred to the web version of the article.)

The crystal structures of GDO from *E. coli* O157:H7 (PDB: 2D40) [100] and from *Silicibacter pomeroyi* (PDB: 3BU7) have been solved [101]. The active sites are superimposable and structures show the metal binding site in the N-terminal cupin domains, which are oriented outwards in the tetrameric structure and thus readily accessible for substrate.^6^ The iron cofactor is coordinated by the ɛ-nitrogen atoms of the three histidine residues (His119, His121, and His160 in *Silicibacter* GDO) and one water molecule. The enzymes were crystallized aerobically and Fe may be present as Fe(III), butit is modeled with Fe(II) in the *S. pomeroy* structure. *E. coli* GDO was strongly inhibited by Cu(II), Zn(II) and Mn(II) [100]. The glutamate moiety in the conserved cupin motif is substituted by an alanine in GDOs, thus presumably providing space for bidentate binding of the substrate and allowing for the sixth coordination site to be free for O_2_ coordination. It has been suggested that the mechanism is supported by acid–base catalysis [100] and, based on in silico docking studies, it has been proposed that His162 acts as proton donor to the evolving peroxidate intermediate (Fig. 17), while the dyad of Asp175 and Gln108 (numbering based on the *S. pomeroy* GDO structure) assists in deprotonation of the 5-hydroxyphenyl group (Fig. 17) [101]. The metal center of *S. pomeroy* GDO with the respective outer shell residues that are suggested to assist in catalysis are shown in Fig. 18.

##### 2.2.3.2 Salicylate 1,2-dioxygenase (SDO)

**Fig. 19:**
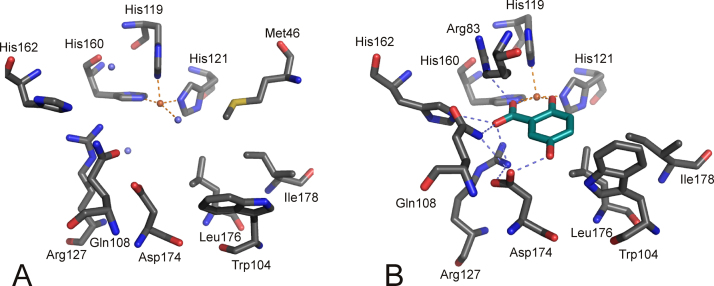
Iron center and substrate binding pocket of SDO from *P. salicylatoxidans* in its (A) free (PDB: 2PHD) and (B) gentisate chelated (PDB: 3NL1) forms. Some residues are omitted for clarity. Oxygen atoms and nitrogen atoms are shown in red and blue, respectively. The substrate ligand is in teal. Water molecules are shown in slate blue. H-bonds are indicated as slate blue dashed lines. Note that substrate binding leads to a movement of the Met46 residue away from the substrate binding site, while Arg83 moves closer above the plane of the substrate ligand. Residues, Gln108, Asp174, His162, Arg127 Trp72 are repositioned due to substrate binding. Further note that residues analogous to Gln108, Asp174, His162 have been suggested to promote acid–base catalysis in GDO. (For interpretation of the references to color in this figure legend, the reader is referred to the web version of the article.)

SDO has been found in a strain that is competent to degrade substituted naphthalenesulfonates, which are used as industrial detergents and building blocks. SDO from *Pseudomonas salicylatoxidans* BN12 has been crystallized and shows the 3-His metal binding center in the N-terminal cupin domain [102]. The fold of SDO is very similar to the previously discussed GDO structures. The enzyme converts gentisate, 5-aminosalicylate, and 1-hydroxy-2-naphthoate with markedly higher activity than salicylate, whereby the ‘best’ substrate gentisate is cleaved at >100-fold efficiency [93]. The enzyme can furthermore convert a range of halogenated salicylates as well as 3-amino- and 3- and 4-hydroxysalicylate [103]. The crystal structures of the anaerobic complexes of SDO with salicylate, gentisate and 1-hydroxy-2-naphthoate have been solved. Principle active site organizations of substrate-bound structures are rather similar and, therefore, will be discussed regarding the gentisate chelated structure of SDO. The substrate bound structure (Fig. 19B) reveals – in comparison with the free enzyme structure (Fig. 19A) – a structural rearrangement from an open to a closed form upon substrate binding. Residues Gln108, His162 and Asp174, which correspond to Gln108, His162 and Asp175 in GDO, have been implicated as acid–base catalysts there. These residues reorient upon substrate binding and form a H-bonding network with the substrate carboxylate and with Arg127, which also repositions. Arg83 enters the 6 Å sphere of the metal ion, which defines the active site, and also H-bonds to the ligand's carboxylate moiety, while Met42 leaves the active site and also Trp104 reorients.

The structures confirm the previously suggested substrate binding mode in GDOs. There are some minor structural deviations between GDOs and SDOs and also between SDOs that have different substrates bound, which will not be discussed in detail here. Most noteworthy, Arg127 and His162 show a substantially different orientation in GDOs and SDOs. Also, in the structures where salicylate or 1-hydroxy-2-naphthoate are bound, which in contrast to gentisate miss a substituent at position 5, the two side chains of Trp104 and Asp174 are involved in a hydrogen bonding network with one water molecule in the cavity, which is absent in the case of the structure that has gentisate bound (not shown). This interaction has been suggested to play a central role in substrate recognition and catalytic intermediate stabilization [104]. Asp174 is highly conserved in all known GDOs as well, while residue 104 is a Tyr residue in most GDO sequences and a serine residue in NDO. The latter cannot cleave gentisate or salicylate [94], [105].

##### 2.2.3.3 Gentisate versus homogentisate dioxygenase mechanism

Although showing a distinct metal binding motif, HGDO catalyzes the analogous reaction to GDO and reactions are believed to proceed via an analogous mechanistic pathway [97], [106], [107]. Generally, HGDO and GDO have strong overall structural similarity [100], [107] as they share the bicupin fold. Yet HGDO, which has the active site located in the C-terminal cupin domain, employs the facial 2-His-1-carboxylate triad for catalysis [107], while GDO and SDO rely on the 3-His center.

**Fig. 20:**
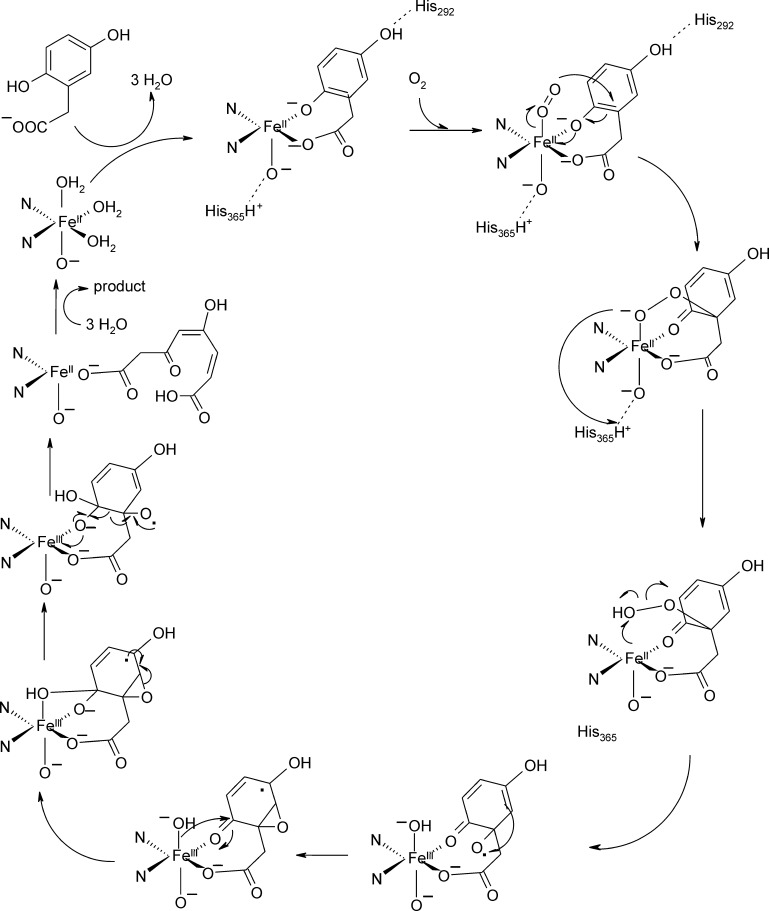
Homogentisate mechanism as deduced from DFT calculations [99], which were calculated in the presence of putative catalytic outer shell residues His365 and His292 [107].

It is not clear why such apparently similar catalysis depends on distinct metal binding centers which are strongly conserved in the respective classes of GDOs/SDOs and HGOs. When considering a putative role of the metal binding motif as a stabilizer of the correct ionization state of the ligand, as proposed based on results from Dke1 [78], a possible explanation may lie in the co-evolution of outer-shell residues. The substrate bound structures of SDOs and GDOs and structural alignments suggest an interaction of aspartate, histidine and, in the case of GDO, tyrosine residues with the substrate/intermediate ligand. The presence of two arginine residues in the active site is demonstrated for SDO. For HGDO, by contrast, two histidine residues and no other charged residues are found in the free HDO crystal structure (no substrate bound structure is available). Titus and coworkers have suggested that His292 and His365 or a metal bound hydroxide may help deprotonate the 5-hydroxy and 2-hydroxy positions (Fig. 20). A reasonable hypothesis is that these active site residues, together with the respective metal center collaborate to keep the substrate ligand in the correct protonation state. However, in light of the major structural rearrangements in SDO upon substrate binding, structural data of substrate-bound proteins will be required to further investigate this hypothesis.

*Computational studies*: More recent quantum-mechanic calculations of a strongly related, albeit 2-His-1-carboxylate-coordinated system, homogentisate 1,2-dioxygenase (HGDO), have suggested a mechanism that is similar to the one proposed for GDO. Calculations suggest that, indeed, formation of the peroxidate intermediate from substrate and O_2_ proceeds without a net change of the metal ions oxidation state. The transition state shows peroxo-character on the dioxygen fragment, while the C1-bound phenolic group turns into a ketone functionality and carbocation character is found at C2. This is in good agreement with the proposed mechanism for GDO (Fig. 17) [97]. However, it was found that a homolytic cleavage of the superoxide's O—O bond is more likely to occur than heterolytic cleavage (previously suggested for the GDO mechanism), which leads to an epoxide-radical intermediate and in several steps finally to the product (Fig. 20). Note that the differences regarding the depicted proposed pathways in Fig. 17 and 20 reflect different suggested hypotheses and not an intrinsically different reaction pathway of HGDOs and GDOs).

### 2.3 3-His-1-carboxylate motif

#### 2.3.1 Quercetin 2,3-dioxygenase (QDO)

##### 2.3.1.1 Enzymology and metal ion promiscuity

QDOs are key enzymes in the assimilation of the plant flavonol quercetin and related structures and have first been reported in *Aspergillus* strains [108], where they are excreted in order to decompose the natural compounds [109]. Beside fungal QDOs, also bacterial QDOs have been described since. QDOs are not strictly Fe(II) dependent and, indeed, *Aspergillus* dioxygenases have generally been considered Cu(II) dependent [110], [111], although alternate metal variants of the enzyme have been reportedly active in metal replacement studies [111].

Bacterial QDOs show varying metal cofactor preferences. *Bacillus subtilis* QDO (BsQDO) produced in *E. coli* [112], [113] has Fe(II) bound. Yet, reconstituted forms of BsQDO with Fe(III), Mn(II), Co(II), Ni(II) or Cu(II) as a cofactor showed similar or higher activities compared to their Fe(II) containing counterpart [114]. It was consequently suggested that the Fe(II) content of recombinant BsQDO may be due to heterologous expression conditions in *E. coli*. Based on catalytic activities, Mn(II), which showed highest activity with a turnover number of 25 s^−1^ was proposed as the native metal ion [115]. A QDO from *Streptomyces* sp. FLA was reported to preferentially incorporate Ni(II) and Co(II) and had the highest turnover number (40.1 s^−1^) with Ni(II) incorporated.

##### 2.3.1.2 Structure and active-site geometry

Structures from *Aspergillus japonicus* [116] and *B. subtilis* show that the Fe(II) form of BsQDO is a homodimer composed of bicupin subunits with each of the resulting four cupin domains binding a metal ion [114]. The overall structure of Cu(II) QDO from *A. japonicus* resembles that of BsQDO except that the fungal dioxygenase is glycosylated at the dimer interface and only its N-terminal cupin is occupied by a metal ion. Also, the substrate-binding site of BsQDO appears to be more accessible to substrate with less bulky side chains. The Fe(II) cofactor of BsQDO is penta-coordinate in each domain and has four protein ligands (3-His-1-Glu) derived from cupin motifs 1 and 2, and one water ligand. The N- and C-terminal metal sites show different geometries (not shown) however, namely a trigonal bipyramidal geometry with a strongly bound glutamate moiety (2.1 Å) in the N-terminus and a distorted tetrahedral geometry with the glutamate side chain positioned more remotely from the metal center (2.4 Å). Similar conformations (close and distant) of the analogous Glu-moiety are found in fungal Cu(II) QDO.

**Fig. 21:**
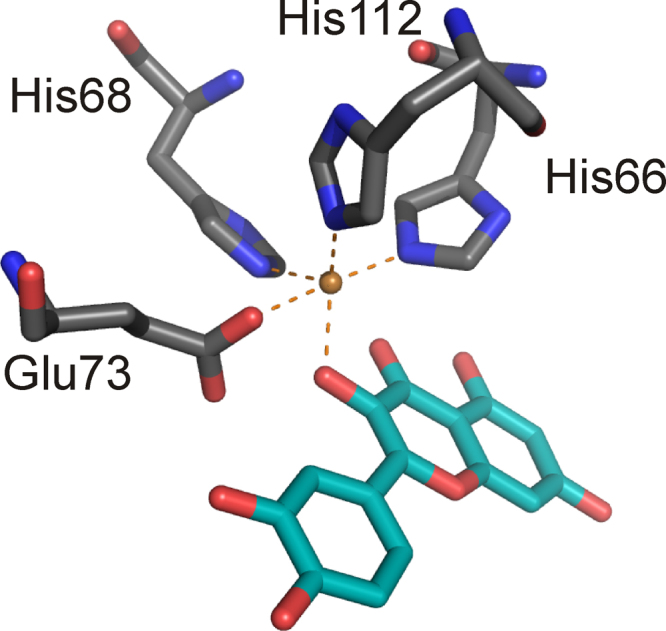
Substrate bound metal center of Cu(II)-QDO from *A. japonicus* (PDB: 1H1I) The first coordination sphere is shown. Oxygen atoms and nitrogen atoms are shown in red and blue, respectively. The substrate ligand is in teal. (For interpretation of the references to color in this figure legend, the reader is referred to the web version of the article.)

A substrate bound, anaerobic crystal structure is only available for Cu(II)-QDO from *A. japonicus* and reveals a mono-dentate coordination of quercetin to Cu(II) (Fig. 21).

The Glu ligand is in the ‘open’ conformation and positioned such that it can interact with the coordinating hydroxy group from quercetin. It has been proposed that the Glu moiety holds the substrate proton in place during catalysis and coordinates the metal ion via its carbonyl group [117]. The substrate shows some distortion, whereby the C2 atom shows apparent sp^3^ hybridization suggesting that this may be due to stabilization of a cation centered radical at C2 [117]. Notably, residues that stabilize the distorted structure are not conserved in the *B. subtilis* structure, which implies that this distortion may not be crucial for catalysis. EPR studies, furthermore, show a Cu(II) species and not a Cu(I)-substrate radical species, as may be expected [118]. Also, Mn(II) and Co(II) oxidation states are observed upon quercetin binding according to EPR spectroscopy [115], [119].

##### 2.3.1.3 Reaction mechanism

**Fig. 22:**
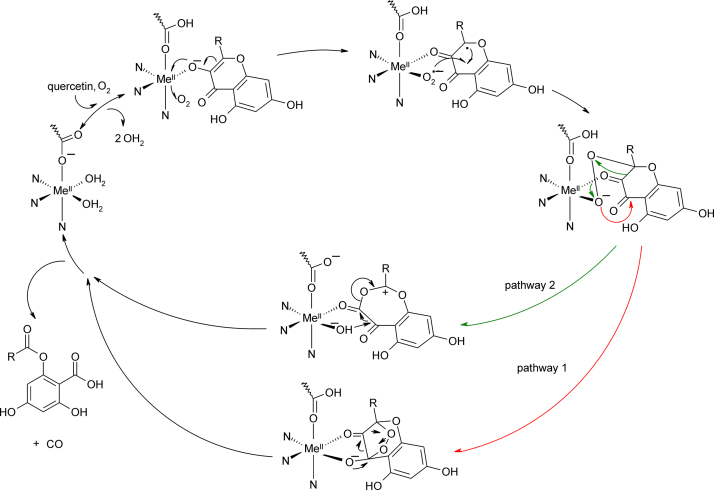
Proposed catalytic pathway of QDOs, consolidated from the mechanistic scenarios suggested for the Mn(II)/Fe(II) enzyme [115] and the Cu(II) enzyme [117]. Two alternative cleavage mechanisms, a nucleophilic ring formation (red, pathway 1) and a Criegee rearrangement (green, pathway 2) are depicted. (The alternative redox-independent dioxygen reduction mechanism, which proceeds via peroxidate formation in one step, is not shown.). (For interpretation of the references to color in this figure legend, the reader is referred to the web version of the article.)

*Aspergillus flavus* QDO incorporates two atoms of oxygen into the substrate upon cleavage with the evolving carbon monoxide remaining unlabeled, as demonstrated by ^18^O incorporation studies [120]. Consequently, a reaction mechanism has been proposed where the C-3 of the quercetin structure is oxidized by molecular oxygen to yield the respective peroxidate (Fig. 22). Substrate cleavage may then proceed via nucleophilic attack (pathway 1) or Criegee rearrangement (pathway 2) [111] of the peroxidate moiety.

Based on structural and EPR data two principle oxygenation mechanisms have been envisioned for QDOs, one that depends on the redox-properties of the metal cofactor and one that does not. In the latter case, O_2_ is reduced by the substrate, whereby the metal ion is not redox active but acts as a Lewis acid and enhances the intrinsic activity of the substrate by stabilizing its anionic form. The analogous non-enzymatic oxygenative quercetin cleavage is long known [121]. A similar reaction is performed by the cofactor independent 1-H-3-hydroxy-oxoquinaldine-2,4-dioxygenase and 1-H-3-hydroxy-oxoquinoline 2,4-dioxygenase, two enzymes that provide a stabilizing oxy-anion hole for generation of a substrate anion, which acts as an electron donor for O_2_ reduction [122]. A comparable mechanism might very well explain the metal ion promiscuity of QDO.

The alternative mechanism involves electron transfer between the metal ion and O_2_ and depends on the direct binding of O_2_ to the metal cofactor. The coordination found in the crystal structure of *A. japonicus* shows quercetin bound in a monodentate mode and thus leaves – beside the 3-His-1-Glu metal binding motif – a vacant site for O_2_ binding.

The pronounced metal ion promiscuity of QDO, which can use Fe(II), Fe(III), Mn(II), Ni(II), Co(II) and Cu(II) efficiently seems to be hard to reconcile with the latter scenario. However, in the case of the cofactor promiscuous 2,3-homoprotocatechuate dioxygenase (2,3-HPCD), a facial triad enzyme, it was suggested that the metal center may act as an electron conduit [123]. Transient Mn(III) and Fe(III) reaction-intermediates have been trapped and characterized in 2,3-HPCD [124], [125] indicating initial one-electron transfer from the divalent metal center to O_2_. It has been argued that a strong coupling of metal ion oxidation and subsequent reduction by the substrate may compensate for one another, so that the net rate of these two steps could be similar for metal ions with distinct redox potentials [123]. Very recently, Kumar and co-workers have successfully used nitrosyl-hydride as an iso-electronic mimic for O_2_ in the quercetin conversion by Mn(II)-BsQDO but not its Co(II)- and Fe(II)-substituted counterparts [126] and it was therefore argued that the described reaction proceeds via a metal mediated electron transfer mechanism. Indeed, the two scenarios may not be mutually exclusive, and in cases such as Ni(II), where the alternative redox state may be hard to access, the cofactor's mechanistic role may actually be that of a Lewis acid. The proposed redox metal dependent reaction mechanism is depicted in Fig. 22.

*Computational analyses:* DFT studies of Siegbahn demonstrate that initial attack of copper by O_2_ is preferable to attack of substrate, whereby a low lying excited state of the Cu(I) substrate-radical species ensures that the copper will remain in its divalent oxidation state. The study furthermore suggests that a nucleophilic attack of C1 by the peroxidate moiety (pathway 1, Fig. 22) is the energetically preferred quercetin cleavage pathway [127].

##### 2.3.1.4 Biomimetic complexes

**Fig. 23:**
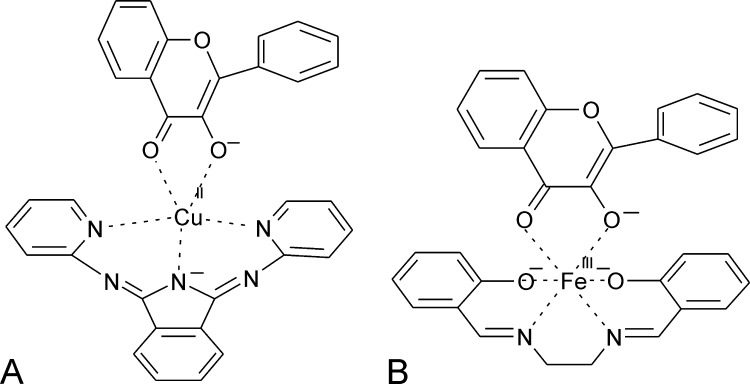
Biomimetic complexes of QDO(A) Cu(II)(fla)(ind) and (B) Fe(III)(fla)(salen) adapted from refs. [128] and [129].

Various potential mimics of the QDO metal center can convert flavonolate (fla) in O_2_-dependent reactions. Two complexes appear to be particularly relevant models for QDOs. (i) A complex of Cu(II) with 1,3-bis(2-pyridylimino)isoindoline (ind) binds the organic ligand in its deprotonated form and chelates the metal ion through three nitrogen atoms. fla coordinates through its keto- and enolate oxygen atoms in the complex, which crystallizes as Cu(II)(fla)(ind)··CH_3_CN (Fig. 23A) [128]. The geometry around Cu(II) is described as trigonally distorted square pyramidal. The complex is converted at temperatures >80 °C in the presence of O_2_ to 2-benzoyloxybenzoate, in analogy to the reactivity of QDOs. Up to seven turnovers of the flavonolate substrate could be achieved, furnishing 2-benzoyloxybenzoate and its hydrolytic degradation products. (ii) An Fe(III)-salen complexed with flavolonate (Fig. 23B), which has been crystallized, shows a distorted octahedral geometry around the Fe(III) center. Again, fla is coordinated as a bidentate ligand and the salen ligand shows a strongly twisted conformation. The complex reacts with O_2_ at measurable rates only at elevated temperatures (100–120 °C). However, in the presence of bulky carboxylate co-ligands reaction rates are dramatically increased, up to 100 fold for a 10-fold excess of triphenylacetate. In the presence of the carboxylate co-ligands, superoxide is formed during the O_2_ dependent conversion. ^18^O labeling studies confirm that, in analogy to quercetin conversion by QDOs, both atoms from molecular oxygen are incorporated into the organic product, while CO remains unlabeled. Consequently it has been suggested that the co-ligands cause mono-dentate binding of the substrate and thus allow direct interaction of O_2_ and the metal cofactor and thus promote the reaction, in accordance with the principle mechanism outlined in Fig. 22 [129].

#### 2.3.2 Aci-reductone dioxygenase (ARD)

##### 2.3.2.1 Physiological significance

Aci-reductone dioxygenase (ARD) has originally been reported as an enzyme of the methionine salvage pathway in *Klebsiella pneumoniae*. This pathway recycles methionine from 5′-methylthioadenosine [130], which is formed during the biosynthesis of polyamines or ethylene. In *Arabidopsis thaliana* it is an effector of the heteromeric G protein β subunit and thus modulates cell division [131]. ARD cleaves 1,2-dihydroxy-5-methylthiopent-1-en-3-one and analogous aci-reductones in an O_2_ dependent reaction [132], [133].

##### 2.3.2.2 Reaction mechanism and metal ion promiscuity

**Fig. 24:**
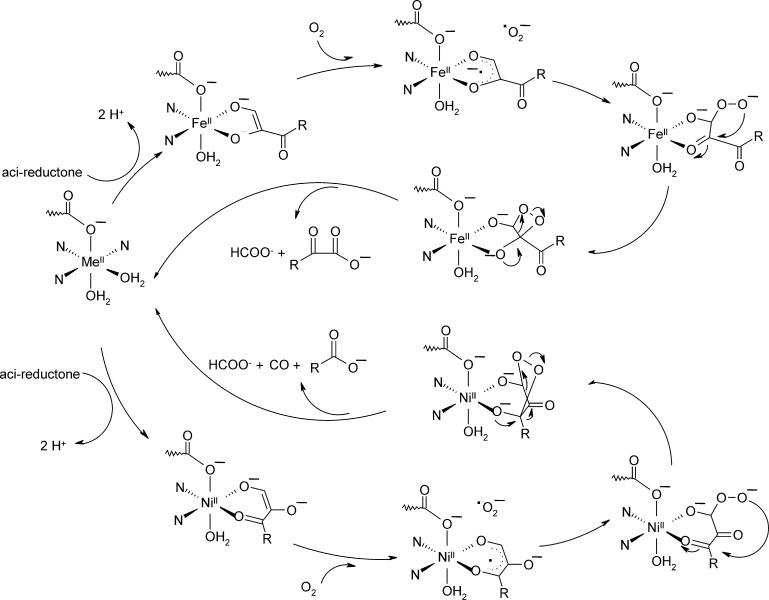
Proposed catalytic pathways for Fe(II)-ARD (upper cycle) and Ni(II)-ARD (lower cycle), adapted from ref. [138].

All available mechanistic studies have focused on ARD from *K. pneumoniae*. The most notable property of ARD from *K. pneumoniae* is its potential to cleave the aci-reductone substrate via two distinct pathways, depending on the incorporated metal cofactor [134]. Fe(II) containing ARD – and with reduced activity also Mg(II) substituted ARD – produces the expected intermediates of the methionine salvage cycle, formic acid and the corresponding oxo-acid (see Fig. 24, reaction products of the upper cycle), while the Ni(II)- or Co(II)-containing versions promote an off-pathway reaction which yields carbon monoxide, formic acid, and the corresponding shortened acid (see Fig. 24, reaction products of the lower cycle). The kinetic parameters of Ni(II)-ARD and Fe(II)-ARD are comparable, with *k*_cat_ values of 500 s^−1^ and 260 s^−1^, respectively, and $K_{{\text{O}}_{2}}$ and *K*_M-aci-reductone_ values of about 50 mM for both enzymes [133]. ^18^O isotope labeling studies have demonstrated that both, the Ni(II) and Fe(II) dependent pathways, proceed via complete incorporation of molecular oxygen into each of the organic products, leaving carbon monoxide unlabeled [132]. Ligand-binding experiments are consistent with an ordered mechanism in which the substrate binds before O_2_. UV spectroscopic studies suggest that substrate is bound as a di-anion in both metallo-isoforms [133]. Spin-trapping experiments with a cyclopropyl substituent bearing substrate analogue as a suicide inhibitor have given inconclusive results.

A mechanism has been proposed, where a single electron transfer from the metal bound di-anionic substrate to O_2_ is followed by a combination of the resulting radicals to form the peroxidate species at C1 [135]. Given that the redox-inactive Mg(II) ion can readily substitute for Fe(II) in ARD, no redox-active role of the metal cofactor is proposed. The peroxidate species then performs a nucleophilic attack at the substrate's C2 (Fe(II)-ARD) or C3 (Ni(II)-ARD), and after electro-cyclic reaction of the resulting four- or five-membered ring, the respective reaction products result (Fig. 24). It has been suggested that the role of the metal ion is to activate the respective carbon for nucleophilic ring closure via Lewis acid interactions with the coordinated oxygen moiety and consequently that differences in substrate coordination geometries in Ni(II)-ARD and Fe(II) ARD may determine the respective catalytic route [135].

##### 2.3.2.3 Enzyme- and metal-center structures of Fe(II)- and Ni(II)-ARD

**Fig. 25:**
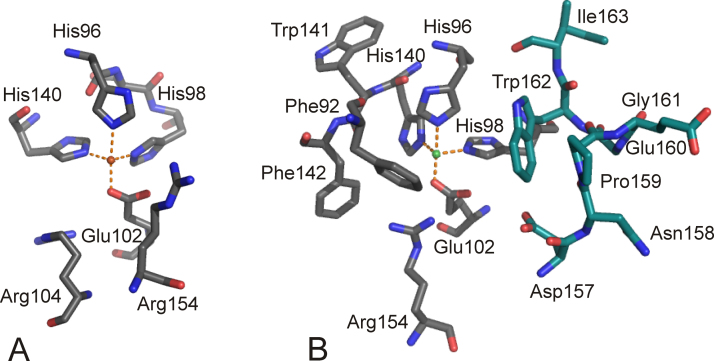
Active site of (A) Fe(II)-ARD (PDB:1HJI) and (B) Ni(II)-ARD (PDB: 1ZZR). The 6 Å sphere surrounding the metal center of a representative NMR structure from the deposited data-set of NMR structures is shown for each variant. Additionally, for Ni(II)-ARD C-terminal residues in the vicinity of the metal center (Asp157-Ile163) are shown in teal. Note that for the Fe(II)-ARD structure the N-terminus is disordered and hence not present in the deposited structure. Generally, the Ni(II) variant appears more tightly packed around the metal center. Fe(II) is shown in orange, Ni(II) is depicted in green. Oxygen atoms and nitrogen atoms are shown in red and blue, respectively. Metal bound waters have been omitted for clarity. (For interpretation of the references to color in this figure legend, the reader is referred to the web version of the article.)

ARD is a monocupin and the overall-structure of Ni(II) ARD was determined by high-resolution NMR [135], [136]. It was later refined by using ^13^C,^15^N double quantum spectroscopy [137] and by modeling of the backbone structure in the vicinity of the metal ion based on the crystal structure of mouse ARD (PDB: 1VR3), which had become available and shows 23% sequence identity with ARD from *K. oxytoca*.^7^ The Fe(II)-structure of ARD was solved indirectly, via the His98→Ser ARD variant, in which the presumable metal binding histidine residue is substituted by serine and no metal cofactor is bound to the protein. The variant is more stable than its wild-type Fe(II)-ARD and apo-ARD counterparts and shows a high structural similarity to Fe(II)-ARD, as determined by comparison of one- and two-dimensional ^1^H NMR and ^1^H ^15^N heteronuclear single quantum coherence (NSQC) spectra of Fe(II)-ARD and the variant [138]. The structures suggest His96, His98, His140 and Glu102 as the metal binding residues. However, as the structure surrounding the paramagnetic metal center (∼9 Å sphere) cannot be determined directly via NMR spectroscopy due to line broadening, Ni(II) and Fe(II) coordination was probed via X-ray absorption spectroscopy (XAS) [139], [140]. For both, the Ni(II) and Fe(II) containing resting ARD, octahedral coordination by N/O was established with 3–4 coordinating histidines, and again, for both isoforms it was suggested based on XAS data that upon substrate binding one histidine may be replaced by water or another amino acid. However, both enzyme variants are strikingly similar in their metal center geometry albeit with somewhat distinct distances of the nitrogen ligands, namely 2.18 Å for Ni(II) and 2.07 Å for Fe(II). Site-directed mutagenesis of the proposed metal binding moieties and of other conserved, putative alternative metal-binding residues in ARD is also in line with His96, His98, Glu102, and His140 being the metal binding residues in both ARD isoforms [140]. However, the overall structures of Ni(II)- and Fe(II)-ARD differ significantly in their C-terminus, which even lead to distinct chromatographic characteristics: In Ni(II)-ARD residues 158–164 form a tight turn, placing Trp162 near the active site (∼7 Å distance from the metal center), while in Fe(II)-ARD the last 22 residues (Asp157-Ala179) are disordered, effectively opening the active site [138] (Fig. 25).

It has been suggested that these structural differences may arise from the slightly different binding geometries or from a switch from Nɛ to Nδ binding of protein ligands at the metal center in the two ARD isoforms, which may transmit the above described perturbations. Note that both coordination modes, binding via Nɛ and Nδ, were equally possible with the Ni(II)-ARD model, whereas modeling a Nδ binding mode severely perturbed the Fe(II)-ARD species. While Ju and co-workers stress that the NMR enzyme models in the vicinity of the metal cofactors should be used with some caution, they point out the interesting possibility that the distinct active site geometries of the structures very well rationalize the distinct reactivities of Ni(II)- versus Fe(II)-ARD. In their substrate-bound models, they demonstrate that in the Fe(II)-ARD form the substrate can coordinate via its adjacent oxygen moieties O1 and O2 without steric restriction, while in the Ni(II)-enzyme the steric interference of Trp162 (Fig. 25) makes O1 and O3 binding sterically more favorable.

##### 2.3.2.4 Biomimetic complexes

**Fig. 26:**
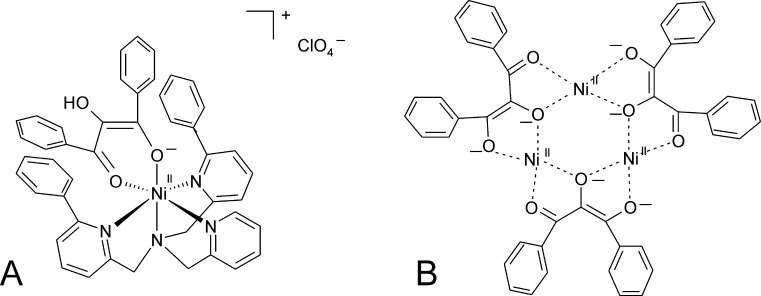
(A) Structure of the Ni(II) (2OH-1,3-Ph_2_PD)(6-Ph_2_TPA) complex as proposed based on ^1^H NMR, electronic absorption and infrared spectroscopy [142]. (B) One layer of the hexanickel enediolate cluster, which is the actual reactive species in the previously described Ni(II)-ARD model [145].

A range of bioinorganic Ni(II)-complexes have been characterized in order to study the enzymatic mechanism of Ni(II)-ARD [141], [142], [143], [144]. One of these complexes showed ARD-type activity and will be discussed here in more detail. No crystal structure was available, but based on ^1^H NMR, electronic absorption and infrared spectroscopy the following structure of the biomimetic complex was proposed: N,N-bis[(6-phenyl-2-pyridyl)methyl]-N-[(2-pyridyl)-methyl]amine (6-Ph_2_TPA) and the aci-reductone analogue 2-hydroxy-1,3-diphenylpropanedione (2OH-1,3-Ph_2_PD) coordinate Ni(II) via the four nitrogen atoms of 6-Ph_2_TPA and via the O1- and O3-moieties of deprotonated (2OH-1,3-Ph_2_PD). The metal center shows octahedral geometry (Fig. 26A). Upon treatment with one equivalent of base and exposure to O_2_ the complex decomposed to one CO and two carboxylate products. ^18^O incorporation studies demonstrated that one atom of molecular oxygen was incorporated into each carboxylate product, in accordance with the enzymatic mechanism of Ni(II)-ARD [142]. Rather recently it was shown that the reactive species in this conversion is a hexanickel enediolate cluster, which was crystallized and forms upon displacement of the chelate ligand 6-Ph_2_TPA [145]. Each hexanickel cluster consists of two layers of Ni(II) centers with each layer having three pseudo-octahedral Ni(II) centers. The deprotonated central oxygen of the enediolate ligand forms the linkage between the layers. Each Ni(II) is consequently coordinated by five oxygen atoms from the enediolate ligands and is capped with an oxygen atom from a coordinated methanol. One layer of the complex is shown in Fig. 26B.

### 2.4 Apocarotenoid-15,15′-oxygenase (AO)

**Fig. 27:**
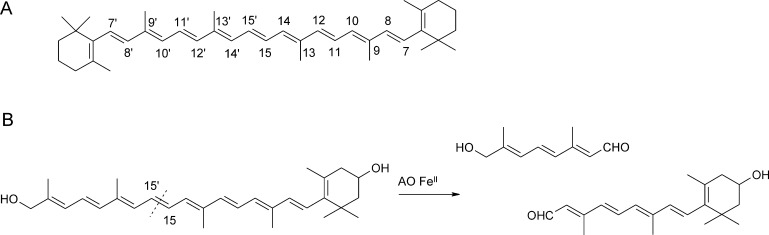
(A) β-Carotene Structure and (B) (3R)-3-hydroxy-8′-apocarotenol cleavage by the structurally characterized AO from *Synechocystis* sp. PCC 6803.

Carotenoids constitute a group of isoprenoid pigments and are generally composed of a C_40_-polyene. Carotenoid oxygenases (CarOs) are found in animals, plants, fungi and cyanobacteria [146], [147], [148], [149], [150], where their cleavage products, apocarotenes, act as physiologically active substances, e.g. as hormones and in signaling pathways. CarOs cleave a range of carotenoids, which are widely distributed in nature. By analogy, apocarotenoid oxygenases convert apocarotenes to a range of biologically active metabolites. CarOs and apocarotene dioxygenases are described as O_2_ and Fe(II)-dependent enzymes and in nature they generally catalyze the double-bond cleavage of carotenoids and apocarotenes, respectively. CarOs with different C-C bond cleavage preferences have been described. β-Carotene along with numbering is shown in Fig. 27A. While β-carotene cleavage dioxygenase I in animals [151], [152], [153] and its analog in fungi [154], [155] cleave the 15,15′ bond of β-carotene to retinal, other CarOs have been reported to regiospecifically cleave the 7,8 (7′8′) [156], 9,10 (9′10′) [157], [158], [159] and 5,6 bonds [160] of carotenoids. Also, lignostilbene structures cleaving oxygenases, which are considered close relatives of CarOs, have been described [161], [162], [163], [164]. CarOs have been classified as both, mono- and dioxygenases based on ^18^O incorporation studies [165], [166], [167]. These studies are generally complicated by a label exchange from the resulting aldehyde with the solvent and, consequently, distinct reaction mechanisms involving epoxide- or dioxetane-formation have been suggested (vide infra, Fig. 29).

**Fig. 28:**
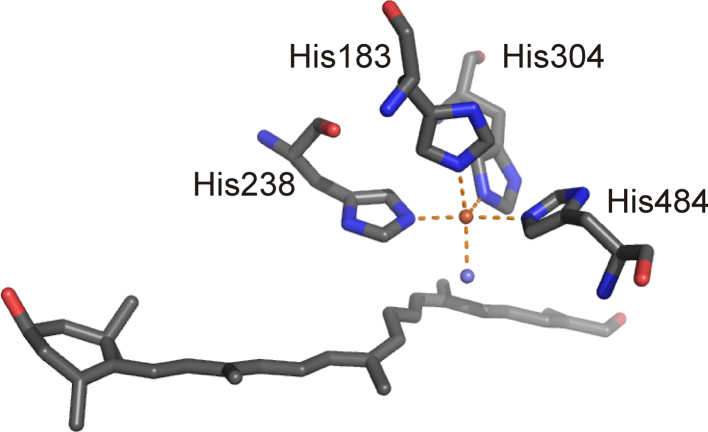
4-His metal binding site of AO (PDB: 2BIW) with bound substrate. Note that the 15-15′ double bond of the apocarotenoid substrate displays cis-configuration. Oxygen atoms and nitrogen atoms are shown in red and blue, respectively. An iron bound water molecule is shown in slate blue. The protein contains Fe(III) in the active site, which is shown in orange. (For interpretation of the references to color in this figure legend, the reader is referred to the web version of the article.)

So far, only the structure of apocarotenoid oxygenase from the cyanobacterium *Synechocystis* sp. PCC6803 [168] has been solved, an enzyme that cleaves (3R)-3-hydroxy-8′-apo-β-carotenol at its 15–15′ bond (Fig. 27B). Notably, the structure was solved in the presence of substrate. The iron center is coordinated via four histidine residues and shows octahedral geometry (Fig. 28).

The apocarotenoid substrate does not coordinate directly to the metal, and leaves one metal coordination site occupied by a water molecule in the enzyme–substrate complex. It should, however, be noted that the structure contains Fe(III) and no information regarding the geometry of the catalytically competent Fe(II) center is available. The substrate, which is located in a non-polar tunnel, could only be fitted into the electron density when the 13–14 and 13′–14′ trans double bonds were changed to cis conformations and it has been proposed that this isomerization, which has an estimated energetic cost of about 100 kJ mol^−1^, may be sterically enforced when the substrate collides with the oxygen ligands of Fe(II) [168]. Based on the structure, it has been proposed that O_2_ might coordinate in a side-on fashion, thus displacing both water molecules upon formation of the ternary complex.

**Fig. 29:**
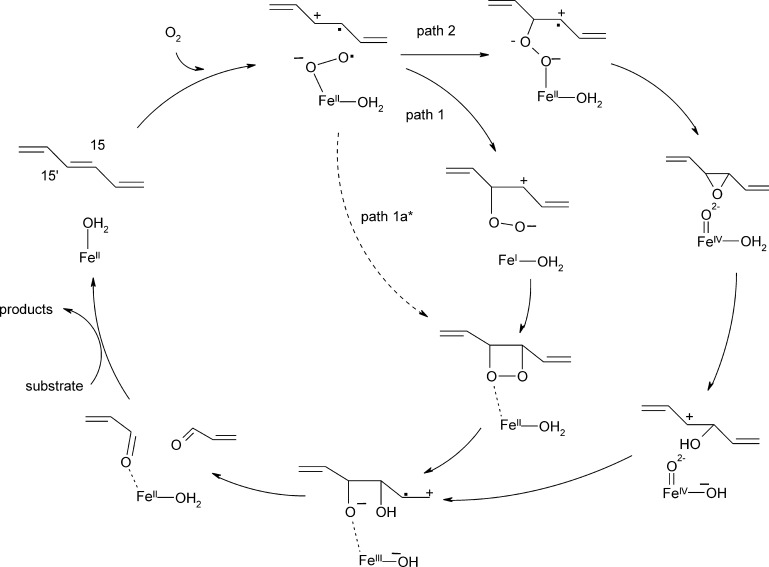
Reaction mechanisms of CarO as proposed based on DFT calculations, adapted from [169]. Only the catalytically relevant substructure of the polyene substrate is shown. The lowest energy pathways found proceed via a dioxetane- (path 1) and an epoxide-intermediate (path 2), respectively. The pathway in the presence of water is depicted. Both reaction pathways have been calculated in the presence and absence of metal-bound water, with similar results, however, in the absence of water the side on addition of O_2_ porceeds via one concerted step (path 1a*, dashed arrow).

*Computational analyses:* A computational study gives insights into probable reaction pathways of AO and, by extension, CarOs, for which very few experimental enzyme-mechanistic studies have been reported so far. In order to investigate the energetic feasibility of the proposed dioxygenase- and monooxygenase-type reactions the respective pathways have been investigated by DFT computational analysis. The presence and absence of a water molecule in the active site have both been considered, as it is not known whether it is present in the catalytically competent Fe(II)-AO complex with substrate. Pathways which were energetically most favorable are outlined in Fig. 29. While Borowski and co-workers found a slight preference for the side-on mechanism in the absence of water (Fig. 29, path 1a*), the energetic differences were small [169], suggesting that mechanisms might be distinct depending on subtle structural differences in the active sites of different CarOs. It has been pointed out that in silico the dioxetane mechanism ensures high specificity, while the epoxide mechanism (Fig. 29, path 2), particularly in the absence of water, would allow for concurring 13′,14′ cleavage.

## 3 Conclusion

The strong conservation of the atypical metal binding motifs necessary for the catalyzed reactions implies that the deviations from the ‘classical’ non-heme-Fe(II) binding site serve a particular purpose in the respective catalytic pathways. For the *2-His-Fe(II) Binding Motif* research of the past few years has impressively demonstrated its rationale: the carboxylate ligand is abandoned in order to provide a site for Cl^−^ coordination, which is subsequently used as a substrate for halogenation. This, however, comes at the expense of a stable Fe(II) center in the absence of the co-factor.

The impact of the *3-His-Fe(II) Center* on catalysis is less well defined. Research shows that the 3-His center provides a stable Fe(II) binding site and for Dke1 it has been shown that it can very well adopt the ‘protective’ 6-coordinate geometry of the resting enzyme in the absence of a stabilizing 1st sphere carboxylate moiety. The unprecedented substrate binding mode and cleavage pathway for phenylpyruvate-type structures at the 3-His center of Dke1 suggest that the metal binding motif may contribute to stabilizing the substrate ligand in the correct ionization state. It is noteworthy that many facial triad enzymes, like catechol 2,3-dioxygenase or α-KG dependent enzymes bind their (co)substrate, which can adopt a mono-or di-anionic form, as a mono-anion. By contrast, for diketones only the mono-anionic state is available upon coordination to the metal cofactor and for CDO the deprotonation of the binding thiol (p*K*_a_ 8.0) and ammonium moieties (p*K*_a_ 10.25) – an ionization state that is generally assumed in QM and QM/MM studies of CDO [62], [63], [170] – could be a requirement for efficient catalysis. For these substrates the 3-His center may be preferable, as it can more efficiently promote ligand deprotonation than the facial triad. A similar rationale may be at work in GDO, however the question remains why HGDO adopts the facial triad coordinated metal center for a rather similar reaction. The answer may lie in the co-evolution of outer-sphere residues, which generally seem of great importance in MNHE functionality. This may also help explain why at least some catechol 2,3-dioxygenase activity is found at the 3-His-Fe(II) center of Dke1. Beside stabilization of the substrate ligand in the correct ionization state, clearly, the substitution of a carboxylate by a histidine ligand will also impact the redox potential and consequently, the primary step of oxygen activation. In Dke1, an apparent decrease of the d-manifold compared to the facial triad coordinated Fe(II) center was observed [74]. Studies of biomimetic complexes of nitric oxide reductase have recently demonstrated how a carboxylate ligand can tune the redox potential of the Fe(II)/Fe(III) couple [171]. While a low redox potential will make the primary reduction of O_2_ more favorable, it may at the same time increase the risk of uncoupled iron oxidation. To understand how this impacts the MNHEs’ ‘choice’ of a particular metal binding motif and to what extent outer sphere residues may counterbalance the impact of the first shell is still a challenging feat and will be crucial when it comes to the design of MNHEs with new functions.

The *3-His-1-Carboxylate-Fe(II) Center* is used by enzymes to convert substrates that are intrinsically susceptible to oxidation, which makes an outer sphere mechanism of electron transfer, that is without direct involvement of the metal cofactor, a plausible possibility. Also a considerable lack of metal specificity is in line with such a mechanism, where the metal cofactor does not directly partake in the reaction. However, a direct interaction of O_2_ and metal center cannot be ruled out on these grounds, particularly for QDO. Indeed, it has been suggested that considerable metal promiscuity may be rather common for dioxygenases that only change the metal ion's oxidation state by one charge during catalysis, as the net rate of the metal ion oxidation by O_2_ and the subsequent reduction by the substrate may compensate one another [124].

Little is known about the function of the *4-His-Fe(II) Center* in carotene dioxygenase. As catalysis does not depend on direct substrate binding, a logical function of the motif may be to shield the active site from water that is not required for catalysis, thus allowing O_2_ and substrate to react.

While nature has demonstrated the feasibility of modulating the metal binding motif in MNHCs in order to tune catalysis, doing so in the context of enzyme engineering is still a great challenge as variation of the metal binding motif typically leads to the destruction of a high affinity Fe(II) binding site. Also, requirements for proper substrate binding and positioning are high. The development of computational tools may help to overcome these challenges in order to fully exploit the catalytic potential of MNHEs.

## Footnotes

1 This review focuses on mononuclear non-heme iron enzymes that utilize O_2_ as a co-substrate. Furthermore, it is restricted to enzymes that use Fe(II) in the resting form of the catalytically competent enzyme. Consequently, lipoxygenases, which show a distinct metal center geometry and depend on the activation of the primary Fe(II) center to Fe(III), which is then the catalytically competent – substrate activating – form of the active site, are not discussed.

2 Deposition number of the bacterial strain at ‘Deutsche Stammsammlung für Mikroorganismen und Zellkulturen’.

3 Notably, in 4-hydroxyphenylpyruvate dioxygenase, which does not depend on an organic co-substrate, the resting metal site is already partially 5 coordinate.

4 Note that very recent molecular dynamic studies suggest that Glu98 is a first-sphere ligand in resting Fe(II)-Dke1, vide supra [73].

5 Note that by analogy two distinct cleavage patterns for phenylpyruvate have previously been reported for oxygenative cleavage of phenylpyruvate in mono- and binuclear Fe(II) complexes, which apparently showed distinct substrate-ionizations [81].

6 In the *S. pomeroyi* structure, the C-terminal cupin domain also has a homologous metal-binding site.

7 Note that the identity of the metal ion cofactor of the mouse protein is not unambiguously determined.
